# Evolutionary history of an Alpine Archaeognath (*Machilis pallida*): Insights from different variant

**DOI:** 10.1002/ece3.10227

**Published:** 2023-07-03

**Authors:** Marlene Haider, Martin P. Schilling, Markus H. Moest, Florian M. Steiner, Birgit C. Schlick‐Steiner, Wolfgang Arthofer

**Affiliations:** ^1^ Department of Ecology, Molecular Ecology Group University of Innsbruck Innsbruck Austria

**Keywords:** evolutionary history, *Machilis*, mitochondrial genome, polyploidy, population genetics, transcriptome

## Abstract

Reconstruction of species histories is a central aspect of evolutionary biology. Patterns of genetic variation within and among populations can be leveraged to elucidate evolutionary processes and demographic histories. However, interpreting genetic signatures and unraveling the contributing processes can be challenging, in particular for non‐model organisms with complex reproductive modes and genome organization. One way forward is the combined consideration of patterns revealed by different molecular markers (nuclear vs. mitochondrial) and types of variants (common vs. rare) that differ in their age, mode, and rate of evolution. Here, we applied this approach to RNAseq data generated for *Machilis pallida* (Archaeognatha), an Alpine jumping bristletail considered parthenogenetic and triploid. We generated de novo transcriptome and mitochondrial assemblies to obtain high‐density data to investigate patterns of mitochondrial and common and rare nuclear variation in 17 *M. pallida* individuals sampled from all known populations. We find that the different variant types capture distinct aspects of the evolutionary history and discuss the observed patterns in the context of parthenogenesis, polyploidy, and survival during glaciation. This study highlights the potential of different variant types to gain insights into evolutionary scenarios even from challenging but often available data and the suitability of *M. pallida* and the genus *Machilis* as a study system for the evolution of sexual strategies and polyploidization during environmental change. We also emphasize the need for further research which will be stimulated and facilitated by these newly generated resources and insights.

## Graphical abstract

The combined consideration of patterns revealed by different molecular markers (nuclear vs. mitochondrial) and types of variants (common vs. rare) that differ in their age, mode, and rate of evolution can provide new insights into species' evolutionary histories. Here, we generated de novo transcriptome and mitochondrial assemblies to apply this approach to *Machilis pallida* (Archaeognatha), an Alpine jumping bristletail considered parthenogenetic and triploid. We highlight the potential of different variant types to unravel complex evolutionary scenarios and the suitability of *M. pallida* and the genus *Machilis* as a study system for the evolution of sexual strategies and polyploidization during environmental change.

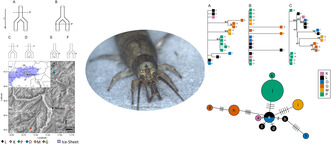

## 1 INTRODUCTION

A central aspect of evolutionary biology is the reconstruction of species and population histories using fossil, morphological, and genetic data. Patterns of genetic variation within and among populations are shaped by evolutionary and demographic processes and carry information that can be leveraged to infer the evolutionary history of natural populations and species. These signatures can be useful to reconstruct population structure, changes in population size, dispersal events, and migration, as well as admixture and selection. Moreover, changes in reproductive mode (e.g. switches from sexual to parthenogenetic reproduction) or genome organization (e.g. polyploidization) leave detectable traces in the genetic makeup of populations and species. However, interpreting these signals and correctly assigning them to the aforementioned processes can be challenging (for animals see, e.g., Jaron et al., 2021). Moreover, polyploidy is frequently coupled with asexuality (Otto & Whitton, 2000), and this combination presents particular challenges for the interpretation of genomic data. Such studies often require high‐quality data, extensive modeling, and simulations as well as accurate estimates of population parameters (e.g. mutation and recombination rate and generation time), which are usually only available for a few intensively studied organisms. Despite excellent theoretical work covering the population genetics of organisms that deviate from sexual reproductive strategies (e.g. Barton & Charlesworth, 1998; Birky, 1996; Charlesworth & Charlesworth, 1997; Felsenstein, 1974; Gerrish & Lenski, 1998; Hill & Robertson, 1966; Muller, 1964) and diploidy (e.g., Burch & Jung, 1993; Dufresne & Hebert, 1994; Maynard Smith, 1978; White, 1973), disentangling the forces that shape genetic diversity remains difficult (Ellegren & Galtier, 2016; Tellier, 2019).

An alternative approach, which is applicable to non‐model organisms as well, is the combined consideration of patterns revealed by different molecular markers and/or types of variants that differ in their age, mode, and rate of evolution. A common practice is the comparison of patterns reflected by nuclear and cytoplasmic molecular markers found in mitochondria (mtDNA) in animals, mitochondria and chloroplasts (ptDNA) in plants, various organelles in fungi and protists, and even intracellular bacteria. Those markers differ from nuclear markers in their effective population size (Ne), recombination as well as mutation rate, and mode of inheritance. For example, mitochondrial and chloroplast markers are subject to stronger drift due to their lower Ne and have therefore frequently been used to investigate recent colonization events and changes in the connectivity among populations (Harrison, 1989; Schönswetter et al., 2005). In addition, incongruence of phylogenetic signals found in nuclear and mtDNA/ptDNA can inform about gene flow events (Avise et al., 1987; Baldo et al., 2011; Funk & Omland, 2003).

When high‐density markers are available, a different approach can be applied that makes use of the fact that patterns of common and rare variants are affected differently by population genetic and demographic processes. On the one hand, common variants are expected to be shared among many individuals and populations and therefore likely reflect broader geographic patterns and older events. On the other hand, many rare variants likely reflect recent mutations that have not yet spread or could be older variants in a migration‐drift ‘quasi‐equilibrium’ (Barton & Slatkin, 1986; Slatkin, 1985; Slatkin & Takahata, 1985; reviewed in Gompert et al., 2014). Regardless of the underlying processes, however, these variants are expected to be spatially restricted under low dispersal and gene flow (Barton & Slatkin, 1986; discussed in Gompert et al., 2014). In fact, several studies found that rare variants reflect more recent and geographically localized processes in humans (Li et al., 2010; Mathieson & McVean, 2012, 2014; Nelson et al., 2012). Moreover, this pattern seems to be congruent in Lycaeides butterflies (Gompert et al., 2014), *Arabidopsis* (Memon et al., 2016), and *Boechera* rockcress (Schilling et al., in prep.) as well.

The aforementioned approaches utilizing rare and common nuclear variants as well as mitochondrial markers can be combined to leverage the differences in resolution and sensitivity to population genetics processes of these three types of variants. We use this highly versatile framework to gain a better understanding of the complex evolutionary history of an Alpine apterygote insect and extend it to reassess previously proposed scenarios of switches in reproductive mode and ploidy (Wachter et al., 2012).

The jumping bristletail genus *Machilis* (Archaeognatha or Microcoryphia) comprises 94 described species and with 55 species, the European Alps harbor the highest species diversity (de Jong et al., 2014; Dejaco et al., 2016). Due to their ancestral winglessness and multiple shared plesiomorphies with other insect orders, they have often been described as ‘ancestral’ or ‘primitive’ (Sturm & Machida, 2001). Given the lack of wings and the high degree of endemism in the genus, these bristletails are assumed to be slow dispersers (Dejaco et al., 2016; Sturm & Machida, 2001), however, modes of dispersal as well as the potential for dispersal are still unknown.

To date, exclusively females have been found for several species and populations despite intensive sampling campaigns (Dejaco et al., 2012, 2016; Janetschek, 1956; Palissa, 1964; Rinnhofer et al., 2012; Sturm & Machida, 2001; Wachter et al., 2012), strongly suggesting the occurrence of parthenogenetic reproduction in the genus. Parthenogenesis is, also in animals, a widespread mode of reproduction (e.g. Bast et al., 2018; Brandt et al., 2017; Liegeois et al., 2020; Magro et al., 2020), and several different modes of transition to parthenogenesis are known, including hybridization, endosymbiont infections, spontaneous mutations, and contagious hybridization (reviewed in Jaron et al., 2021). Genomic features associated with these transitions can include changes in heterozygosity, less effective positive selection, or a low transposable element load. However, these features are not universally applicable and often lineage‐specific (Bast et al., 2018; Jaron et al., 2021, 2022). Karyotyping and flow cytometry results from *Machilis* indicate various instances of polyploidy (Gassner et al., 2014). Interestingly, polyploid animals tend to occur more often at higher latitudes (Lorch et al., 2016) with the vast majority, 80.5% of polyploid species, being found outside the tropics (David, 2022). This trend is particularly pronounced in insects, as 97% of polyploid species are found outside tropical regions (David, 2022). Moreover, glaciation is a significant environmental factor that promotes polyploidy and is of particular importance for insects and amphibians (David, 2022). Polyploidization and asexual reproduction may have played a role in the successful colonization of areas previously covered by glaciers (Lorch et al., 2016). Although polyploidization is indeed strongly correlated with parthenogenetic reproduction in animals, this is not a general rule (Otto & Whitton, 2000), and there is also no such strict association in *Machilis*. In the species studied so far, sexuals were found to be diploid whereas asexuals were classified as either diploid or triploid (Gassner et al., 2014).

The high number of *Machilis* endemics and their distribution in and around the European Alps has sparked interest in their distribution and survival during the ice ages. Identifying and characterizing the different refugia during the last glacial maximum (LGM; 18,000 years before present) (van Husen, 1997) enables a better understanding of current species distributions and levels of diversity, both in terms of species numbers and genetic variation (Holderegger & Thiel‐Egenter, 2009; Knowles, 2000; Schneeweiss & Schönswetter, 2011; Schönswetter et al., 2002, 2005; Stehlik, 2003; Westergaard et al., 2011).

Such information also increases our knowledge on how species can react to climate change and which biological features are associated with successful survival in a changing environment. One open debate in this context revolves around the question of whether and how frequently arctic and high alpine species survived glaciation on nunataks (isolated mountain top areas protruding above the ice sheet) versus in peripheral areas (Schneeweiss & Schönswetter, 2011; Schönswetter et al., 2005). While several studies in plants suggest nunatak survival (Abbott & Brochmann, 2003; Bettin et al., 2007; Parisod & Besnard, 2007; Stehlik et al., 2002; Westergaard et al., 2011), there is little evidence in animals, with the exception of *Trechus* ground beetles from peripheral nunataks in the Orobian Alps (Lohse et al., 2011).

One case in animals, for which central nunatak survival has been suggested (Wachter et al., 2012) is *Machilis pallida* (Janetschek, 1949), an endemic bristletail in the Eastern Alps, residing exclusively on carbonate rock scree above 2000 m above sea level (a.s.l.) (Dejaco et al., 2012; Rinnhofer et al., 2012; Wachter et al., 2012). Based on mitochondrial and AFLP data for three populations, Wachter et al. (2012) proposed that this species survived LGM on both peripheral and central nunataks, indicating that central refugia may be more important than previously thought, and they suggest a complex evolutionary history. Further, the species is considered to be parthenogenetic (Rinnhofer et al., 2012; Wachter et al., 2012) and triploid (Gassner et al., 2014).

Their early divergence in the insect phylogenetic tree, the occurrence of different reproductive systems and ploidy levels, and their patterns of distribution make bristletails in the genus *Machilis* an interesting study object in ecology and evolution. Here, we provide novel genomic resources for this system by presenting the first de novo assembled transcriptome obtained from 17 triploid individuals of *M. pallida* from six populations in the European Alps as well as a de novo assembled mitochondrial genome. We then outline our approach to obtain information on the population structure and biology of this Alpine bristletail species from the generated RNAseq data. Similar RNAseq data sets are available for many non‐model species and while their analysis arguably can pose some challenges, they are also an underappreciated source of information that can be exploited to gain first insights into the evolutionary history of a species and inform the design of targeted follow‐up studies (Feng et al., 2023; Mossion et al., 2022; Thorstensen et al., 2021). Here, we utilize our new resources to combine mitochondrial variation as well as common and rare nuclear variation and assess the distribution of differences and similarities in genetic signatures revealed by those sets of markers to better understand the evolutionary history of this species. Lastly, we discuss our findings in the context of current hypotheses on the reproductive mode, ploidy, and distribution of *M. pallida*.

## 2 MATERIALS AND METHODS

### 2.1 Sample collection

**FIGURE 1:**
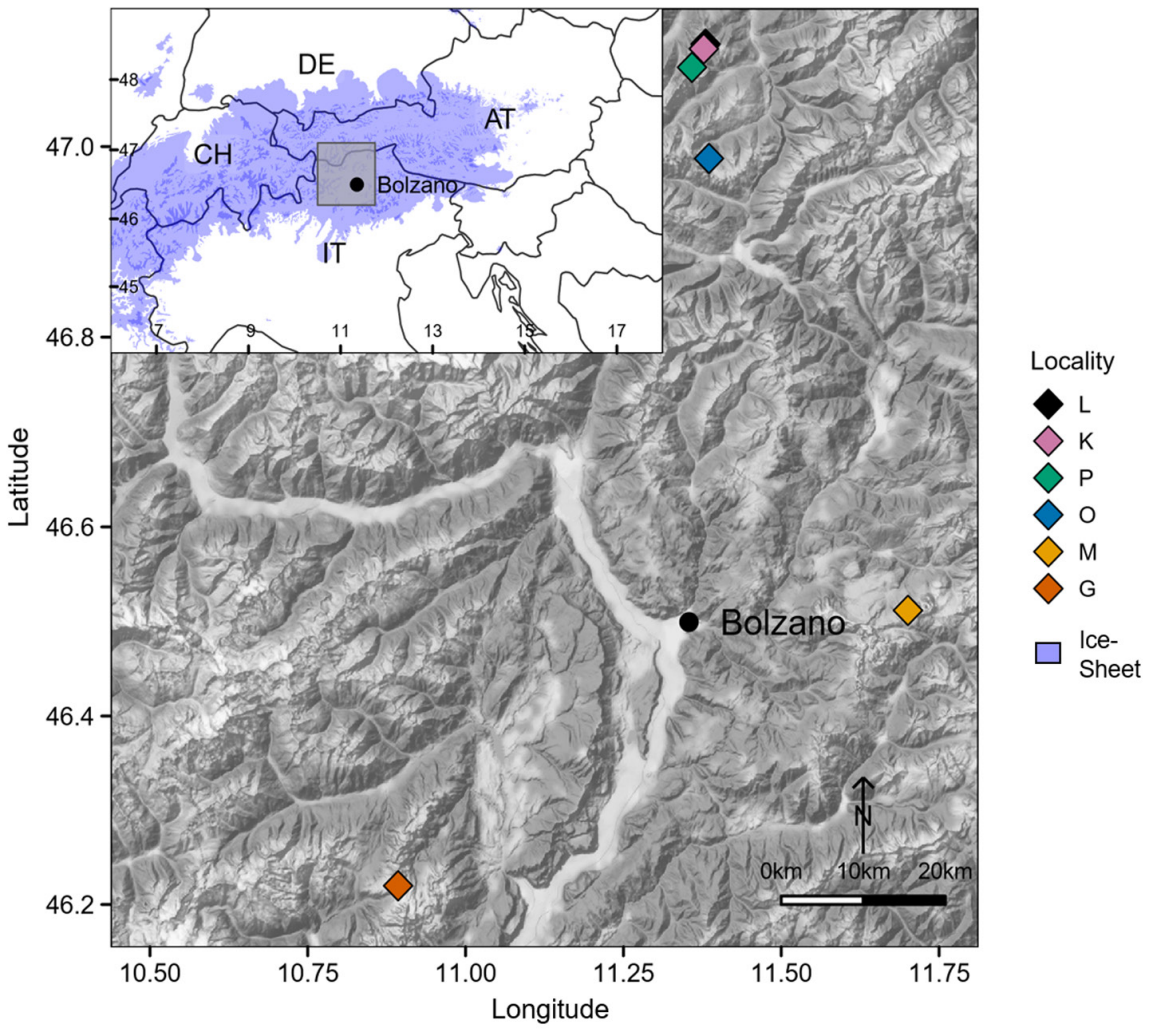
The six sampling locations of *Machilis pallida*. Colors of diamonds correspond to the different populations; Laempermahdspitze (L, black), Kesselspitze (K, pink), Padasterjochhaus (P, green), Obernberger Tribulaun (O, blue), Murmeltierhuette (M, yellow) and Grosté Seilbahn Bergstation (G, orange). The overview map shows the extent of the Alpine glaciers during the last glacial maximum.

We collected 17 adult female (the only sex found so far) *M. pallida* specimens at six locations in the eastern Alps (Laempermahdspitze (L, *n* = 4 individuals), Kesselspitze (K, *n* = 1), Padasterjochhaus (P, *n* = 5), Obernberger Tribulaun (O, *n* = 2), Murmeltierhuette (M, *n* = 2) and Grosté Seilbahn Bergstation (G, *n* = 3)) in September 2016 (Figure 1, Table 1, Table A1). This sampling design includes putative central nunatak populations (L, K, P, O) and putative peripheral nunatak populations (M and G) (see Dejaco et al., 2016; Gassner et al., 2014; Wachter et al., 2012). All individuals were transported alive to the Department of Ecology at the University of Innsbruck, Austria, and kept in plastic boxes supplemented with humid gravel from the collection site at 10°C and a 12:12 h light:dark cycle for 4 days to normalize gene expression. The individuals were identified based on morphological characters using the key in Dejaco et al. (2012) and to ensure that only adults were included only large‐bodied individuals with a fully developed ovipositor were collected.

**TABLE 1:** Sample information. Locality abbreviations correspond to Laempermahdspitze (L), Kesselspitze (K), Padasterjochhaus (P), Obernberger Tribulaun (O), Murmeltierhuette (M) and Grosté Seilbahn Bergstation (G).

ID	Individual	Locality	Longitude	Latitude	Country	Elevation (m a.s.l.)
1	92,360	K	11.377325	47.102333	AT	2280
2	92,361	L	11.378703	47.107025	AT	2220
3	92,362	L	11.379536	47.108	AT	2220
4	92,363	L	11.379769	47.10695	AT	2220
5	92,364	L	11.380089	47.107153	AT	2220
6	92,365	O	11.385581	46.987497	AT	2000
7	92,366	O	11.385383	46.987583	AT	2000
8	92,367	G	10.893125	46.219956	IT	2400
9	92,368	G	10.889464	46.222336	IT	2400
10	92,369	G	10.892083	46.220806	IT	2400
11	92,370	M	11.700775	46.511847	IT	2200
12	92,371	M	11.699558	46.511008	IT	2200
13	92,372	P	11.358669	47.082906	AT	2320
14	92,373	P	11.358606	47.083008	AT	2320
15	92,374	P	11.358481	47.082889	AT	2320
16	92,375	P	11.358458	47.082933	AT	2320
17	92,376	P	11.358381	47.082917	AT	2320

### 2.2 RNA extraction, library preparation, and sequencing

Individuals were shock‐frozen in liquid nitrogen and we performed RNA extraction using the Macherey and Nagel Nucleospin RNA kit following the instructions of the manufacturer. Library construction and sequencing were performed by IGATech. We then constructed a barcoded library for all individuals with the Illumina TruSeq Stranded mRNA Library prep kit following the manufacturer's instructions. Libraries were then sequenced on an Illumina HiSeq 2500 platform in 250 bp paired‐end rapid run mode.

### 2.3 Transcriptome assembly and mitochondrial reads

We performed quality control of the raw data with FastQC (Andrews, 2010) before concatenating raw reads from the 17 individuals. BBtools v37.36 (Bushnell, 2021) was used for (i) quality trimming and quality filtering (bbduk.sh with a minimum length of 10), (ii) decontamination (with bbduk.sh and reference phix174_ill.ref.fa, *k*‐mer size of 31 and hamming distance of 1), and (iii) read normalization (with bbnorm.sh and read coverage of 60 and minimum depth of 5). Quality‐trimmed and normalized reads were assembled using Trinity v2.2 (Grabherr et al., 2011) with the following settings: strand‐specific RNA‐Seq read orientation, minimum contig length of 500, Jaccard clip, and normalization of reads. After assembly, we performed cleaning steps to remove contaminants and redundancy in the transcriptome: (i) Blobtools v1.0 (Kumar et al., 2013) was used for contaminant filtering with two Blastn (Altschul et al., 1990) *e*‐value cutoffs of 1e−3 and 1e−5 to identify contaminants. In this step, transcripts of viruses, archaea, bacteria, and fungi were removed from the dataset. (ii) CD‐HIT‐EST v2 (Fu et al., 2012) was used to find unigenes (options: alignment coverage of 0.9, word length of 8, and cluster to most similar cluster (g) of 1). When multiple transcripts had the same BLAST hit, only the longest was retained. After each cleaning step, a completeness check was performed using Universal Single‐Copy Orthologs (BUSCO) software v3 (Simão et al., 2015). Further, we calculated Nx, ExN50 statistics, and the percentage of raw reads using Trinity v2.2 and we estimated read abundance with the package RSEM (Li & Dewey, 2011). Finally, we aligned individual raw reads to the assembled transcriptome using Bowtie2 (Langmead & Salzberg, 2012) and calculated the coverage using Qualimap2 (Okonechnikov et al., 2016). With Transdecoder v2.0.1 (Haas et al., 2013), possible open reading frames (ORFs) were detected. With these predictions, the annotation with Trinotate v3.1.1 (Bryant et al., 2017) was performed by doing a homology search in BLAST (Altschul et al., 1990) and Swissprot (The UniProt Consortium, 2017), protein domain identification with HMMER v3 (Finn et al., 2011) and PFAM v31.0 (Finn et al., 2014), protein signal peptide prediction with signalP v4.1 (Petersen et al., 2011), transmembrane domain prediction with tmHMM v2.0c (Krogh et al., 2001) and further annotation databases (eggnogg (Huerta‐Cepas et al., 2016), Gene Ontology (GO; Ashburner et al., 2000), and Kegg (Kanehisa et al., 2012)). GO terms per gene were visualized with Web Gene Ontology Annotation Plot v2.0 (WEGO; Ye et al., 2006).

We further created a de novo mitochondrial assembly of *M. pallida* using MITObim (Hahn et al., 2013), which employs a mitochondrial baiting and iterative mapping approach using the MIRA assembler (Chevreux et al., 1999). We used a kmer length of 31 for bait fishing with mirabait using genome skimming data (individual 92,010, Murmeltierhuette, ERS4357532; SAMEA6593248; T. Dejaco, unpubl.) and the *Songmachilis xinxiangensis* mitochondrion as baiting sequence (He et al., 2013) (with MIRA v4.0.2 (Chevreux et al., 1999)) with 30 iterations. We used ORFfinder to search for insect mitochondrial ORFs in the mitochondrial contig, and from the resulting ORFs, we blasted the 10 longest hits using smartBLAST (NCBI, 2021). We also annotated the assembled mitochondrion using MITOS (Bernt et al., 2013).

### 2.4 Alignment and variant calling

We used bbmap (Bushnell, 2021) to align the trimmed and decontaminated reads of all 17 individuals with a minimum identity score of 0.97 for the alignments to the de novo assembled transcriptome and a minimum identity score of 0.90 for the alignments to the mitochondrion. After removing unmapped reads for both nuclear and mitochondrial alignments with samtools v1.9 (Li et al., 2009), we marked duplicate reads with Picard v2.19.1 (Broad Institute, 2018). For the nuclear RNA, we called variants with GATK v3.8 and the UnifiedGenotyper tool (McKenna et al., 2010). Specifically, for nuclear RNA reads, we called variants as triploid, with a minimum phred‐scaled confidence threshold for variants to be called of 50, three alternative alleles, and the SNP genotype likelihood model. For the mitochondrial reads, we used bbmap to call variants with quality score recalibration (bbvarMT.sh) and a ploidy of two. After calling variants, we filtered both the nuclear and mitochondrial variants with a minimum coverage of 128, a minimum mapping quality of 50, and a minimum occurrence of four sequences with the alternative allele. Additionally, we only kept substitutions that were not fixed for either the reference or alternative allele. After filtering the variants, we split the nuclear variants into common and rare variants, where variants with allele frequency <0.1 were considered rare.

### 2.5 Population genetics, haplotype networks, and Neighbor‐Joining trees

We extracted genotypes for mitochondrial variants, as well as common and rare variants using custom python scripts (mafFltr.py). We converted the genotypes of all three variant types into Nexus format (vcf2nex.py) to compute distance matrices and obtain Neighbor‐Joining (NJ) trees for all three variant types in R using the ape package (Paradis & Schliep, 2019), and to obtain haplotype networks for rare and mitochondrial variants using the pegas package (Paradis, 2010). We further calculated Hamming distances from the diploid (mt) and triploid (common and rare) variants for Multidimensional Scaling (hereafter referred to as Principal Coordinates Analysis, PCoA) in R (R Core Team, 2020). Additionally, we calculated pairwise distances among populations and individuals using Nei's genetic distance for both mitochondrial and nuclear variants in R using the StAMPP package (Pembleton et al., 2013).

## 3 RESULTS

### 3.1 Transcriptome assembly and mitochondrial reads

The mean read number per individual after quality trimming, decontamination, and normalization amounted to 8 million (M) reads (SD = 1.7 M) (see Table A1), resulting in a total of 359 M reads used for the assembly. The mean coverage and GC content per individual was 8.6 (SD = 1.8) and 41.1% (SD = 0.9%), respectively (Table A1). After quality filtering, decontamination, and normalization, 46,748,840 reads were used for the assembly. In total, 289,342 contigs (Trinity transcripts) and 117,970 genes (Trinity genes) were assembled. After the removal of redundancy in the dataset, the final set contained 159,192 contigs and 106,399 genes. GC content was 40.39% (Table 1), N50 contig length 1959 bp, and E80N50 contig length 2535 bp. The overall read alignment was 80.07%, of which 68.95% were properly paired. BUSCO revealed a high completeness of the transcriptome (93.3%) when probing the insect database.

In the 159,192 transcripts, 124,989 ORFs were detected. Overall, 61,186 transcripts (64% of all assembled transcripts) were annotated. Of these, 50% were assigned to a unique protein in UniprotKB using Blastx. With Blastp, 38% of the transcripts showed a protein hit. In Eggnog, Kegg, and Blast GO terms, 34%, 35%, and 43% of the transcripts showed a hit, respectively. A total of 65,782 GO terms were assigned to the transcripts. Of these, approximately 32% were described by the aspect biological process, 34% by cellular component, and 34% by their molecular function (Table A2, https://github.com/mphaider/M.pallida.git).

In the *M. pallida* mitochondrial genome assembly, MITObim reached a stationary read number of 54,161 reads after 25 iterations of baiting and mapping, with a length of 15,836 bp for the resulting mitochondrial contig. The 10 longest ORFs on the mitochondrion and annotated protein and RNA coding genes are summarized in Tables A3 and A4, respectively.

### 3.2 Alignment and variant calling

Across the 17 *M. pallida* individuals, the mean number of reads amounted to 40.97 M (SD = 8.7 M) after quality trimming and decontamination. Reads mapped with an average rate of 69.67% (SD = 1.46%) and 7.01% (SD = 2.15%) to the nuclear and the mitochondrial assembly, respectively. After variant calling and filtering, we found a total of 213,321 variants (from 1,196,769 unfiltered variants) using the nuclear assembly as reference, of which 201,195 variants were common and 12,126 variants were rare (i.e. 5.69% of variants were lower than an allele frequency of 0.1). For the mitochondrial alignments, we found 29 variants after filtering (with 32 unfiltered variants).

### 3.3 Population genetics, haplotype networks, and Neighbor‐Joining trees

**FIGURE 2:**
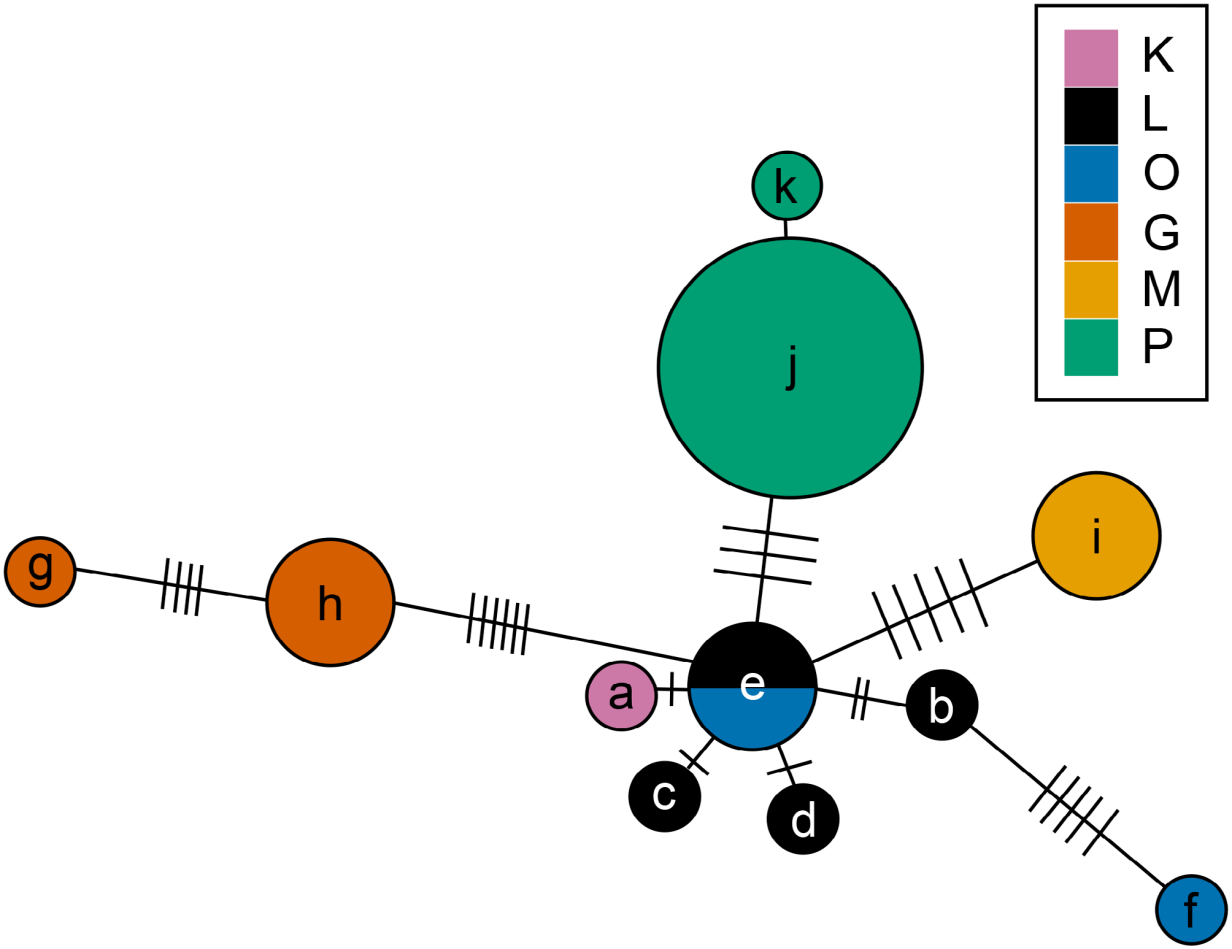
Haplotype network for mitochondrial variants (*n* = 29). Note that a link length of two between haplotypes a and d was omitted in this figure. Colors correspond to the different populations; Laempermahdspitze (L, black), Kesselspitze (K, pink), Padasterjochhaus (P, green), Obernberger Tribulaun (O, blue), Murmeltierhuette (M, yellow) and Grosté Seilbahn Bergstation (G, orange). Lowercase letters a to k denote the 11 mitochondrial haplotypes.

**FIGURE A1:**
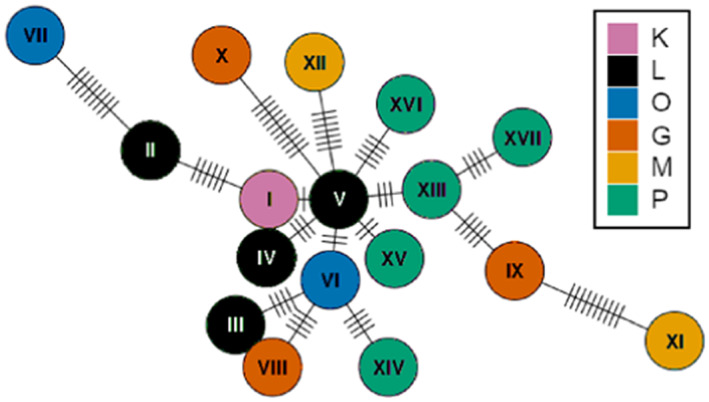
Haplotype network based on rare variants for 17 individuals and haplotypes Colors correspond to the different populations; Kesselspitze (K, pink), Laempermahdspitze (L, black), Obernberger Tribulaun (O, blue), Grosté Seilbahn Bergstation (G, orange), Murmeltierhuette (M, yellow), and Padasterjochhaus (P, green).

In the mitochondrial haplotype network, we found 11 haplotypes (Figure 2), where the two Southern localities (G and M with haplotypes g, h, and i) both split off from a central batch, containing one haplotype each from L and O (e), where link lengths to the Southern haplotypes consist of at least five steps. All remaining haplotypes fork off from the central haplotype (e), with three more haplotypes for L (b, c, and d), one for K (a), two for P (j and k), and one more haplotype for O (f), which links through a haplotype from L (b). A haplotype network for rare variants showing 17 haplotypes for 17 individuals can be found in the Appendix 1 (Figure A1).

**FIGURE 3:**
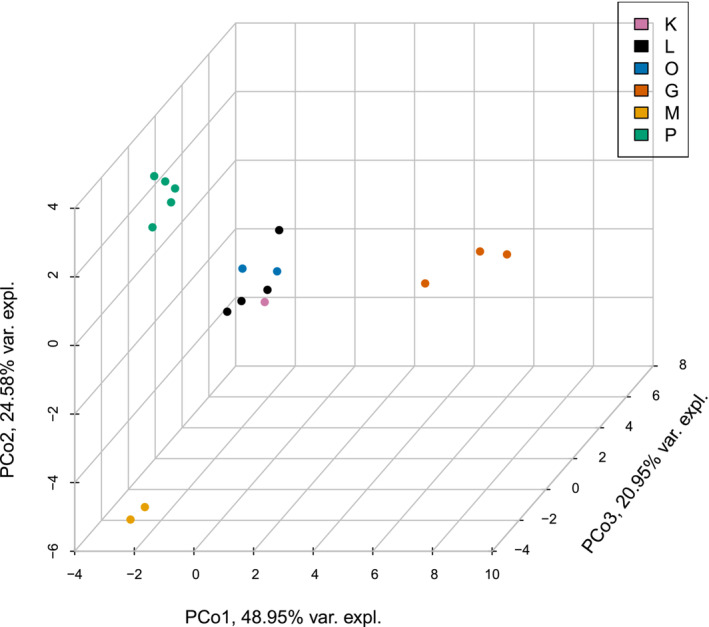
Principal coordinate analysis of 29 mitochondrial variants. Colors correspond to the different populations; Laempermahdspitze (L, black), Kesselspitze (K, pink), Padasterjochhaus (P, green), Obernberger Tribulaun (O, blue), Murmeltierhuette (M, yellow) and Grosté Seilbahn Bergstation (G, orange).

**FIGURE 4:**
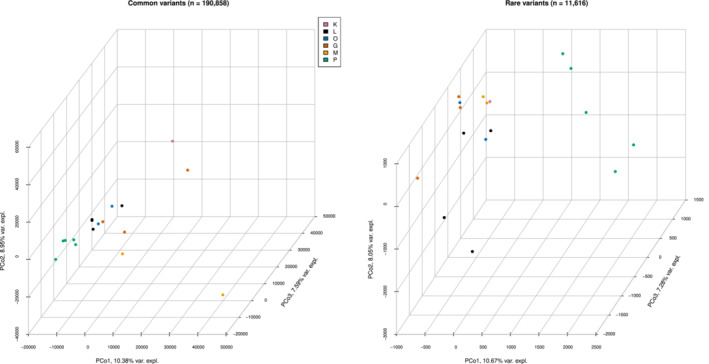
Principal coordinate analysis of common (left panel) and rare (right panel) variants with 190,858 and 11,616 variants. Colors correspond to the different populations; Laempermahdspitze (L, black), Kesselspitze (K, pink), Padasterjochhaus (P, green), Obernberger Tribulaun (O, blue), Murmeltierhuette (M, yellow) and Grosté Seilbahn Bergstation (G, orange).

**FIGURE A2:**
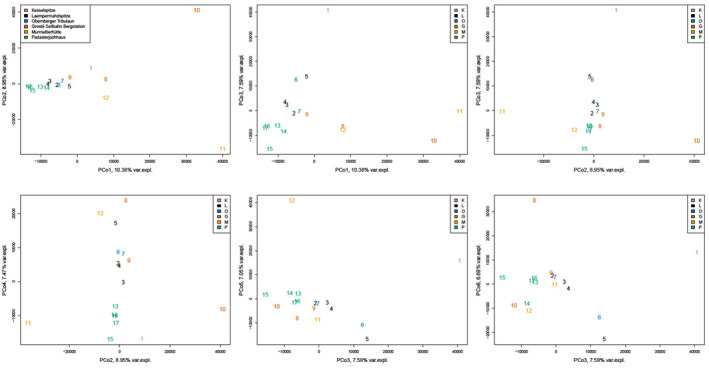
Principal coordinate analysis of 29 mitochondrial variants. Combinations of the first six principal coordinate axes are shown. Colors correspond to the different populations; Kesselspitze (K, pink), Laempermahdspitze (L, black), Obernberger Tribulaun (O, blue), Grosté Seilbahn Bergstation (G, orange), Murmeltierhuette (M, yellow), and Padasterjochhaus (P, green).

Mitochondrial variants were relatively well‐resolved in the PCoA, where the three highest Principal Coordinates (PCos) explained 94.5% of the overall variance across the 29 variants (Figure 3, Figure A2). We found a tight central cluster formed by individuals from O, L, and K, which was adjacent to all individuals from the P site. As shown in Figure 4, M and G locations are placed farthest from the central cluster, and they are positioned on opposite sides of the central cluster (i.e. the two southern locations (M and G) are both closer to the central cluster than to each other, differentiated on all three PCos).

**FIGURE A3:**
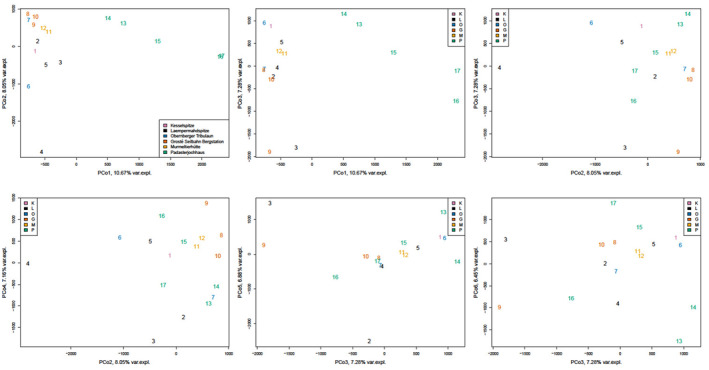
Principal coordinate analysis of 190,858 common nuclear variants. Combinations of the first six principal coordinate axes are shown. Colors correspond to the different populations; Kesselspitze (K, pink), Laempermahdspitze (L, black), Obernberger Tribulaun (O, blue), Grosté Seilbahn Bergstation (G, orange), Murmeltierhuette (M, yellow), and Padasterjochhaus (P, green).

The first three PCos for 190,858 common nuclear variants explained about 27% of the overall variance found and are shown in Figure 4 (see also Figure A3). Here, we also see a tight central cluster, formed by the sites of L, O, two samples from G as well as one individual from M. Close by, P again forms a distinct cluster, yet close to the central group (mainly distinguished on PCo 1 and PCo 3). The remaining M individual is far removed (distinguished mainly on PCo 1), and the last two individuals (from K and G) are somewhat close to each other, yet far removed from the rest, distinguished mainly on PCos 1 and 3. Note that PCos 1 and 3 share patterns, with PCo 2 seemingly differing based on the variance encountered.

**FIGURE A4:**
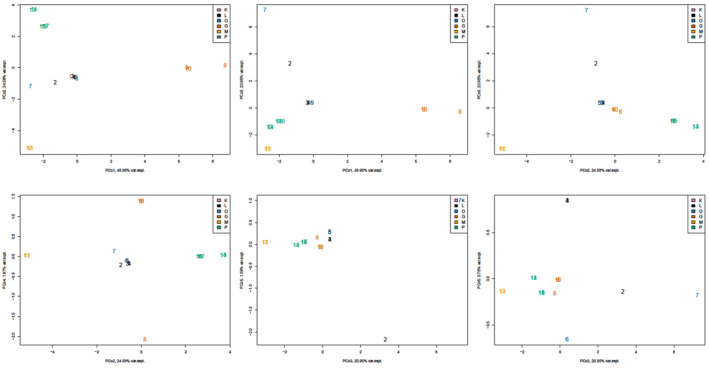
Principal coordinate analysis of 11,616 rare nuclear variants. Combinations of the first six principal coordinate axes are shown. Colors correspond to the different populations; Kesselspitze (K, pink), Laempermahdspitze (L, black), Obernberger Tribulaun (O, blue), Grosté Seilbahn Bergstation (G, orange), Murmeltierhuette (M, yellow), and Padasterjochhaus (P, green).

Principal coordinates of 11,616 rare nuclear variants explained about 26% of the overall variance across the 17 individuals. Figure 4 and Figure A4 depict a cluster, formed by K, M, two samples from G, and two individuals from L. The remaining individual from G is close to the remaining individuals from G on PCo 1 and 2 but different on PCo 3. The remaining 2 L individuals were predominantly distinguished on PCos 2 and 3. Further, variants of P spread the five individuals mainly across PCos 1 and 3.

The resolution of the neighbor‐joining trees varied substantially among the three variant types. For the 29 mitochondrial variants, the Northern locality of P falls into one polytomous group, and so do the Southern localities of M and G as shown in Figure 3. The remaining individuals, from localities L, K, and O, are not resolved. The 12,126 rare nuclear variants do not resolve the relationships among the individuals and we observe a single polytomous block. The highest level of resolution, however, was achieved with the 201,105 common variants, revealing a clear distinction into localities G and M, the central group of L, O, and K, and finally the polytomous individuals from locality P.

Calculated genetic distances largely reflected the results of the Neighbor‐Joining trees and the haplotype network. Nei's distance for the individuals showed different values depending on the variants used. For mitochondrial variants, individuals 5 and 6 from locality L and O, respectively, individuals 9 and 10 from locality G, individuals 11 and 12 from locality M, and individuals 13, 15, 16, and 17 from locality P were genetically identical (Table A5). Individual 14 of P had a greater genetic distance from the other individuals of P (mean distance = 0.035). When considering nuclear variants, all observed distances exceeded 0.05, revealing larger genetic distances between individuals. Additionally, the genetic distances among populations also indicated higher values for mitochondrial variants compared to nuclear variants (Table A6). The southern localities M and G showed the highest values for the genetic distance to the other populations and to each other, and P again shows greater genetic distance to the other northern populations. The largest genetic distance between two localities was found for M and G with 0.467 in the mitochondrial variants and the largest distance for nuclear variants was 0.069 between locality K and M.

## 4 DISCUSSION

This work contributes to ongoing efforts directed toward a better understanding of the complex evolutionary history of Alpine bristletails. For this purpose, we established new resources, particularly novel transcriptome, and mitochondrial assemblies for *M. pallida* Janetschek, 1949. Together with various genotyping‐by‐sequencing approaches, transcriptomic and mitochondrial sequence data can be utilized as cost‐effective options for assessing patterns of population structure and genetic diversity (Hahn et al., 2013; Hirsch et al., 2014), in particular in non‐model organisms where a reference genome is absent and/or genomes are very large (i.e. >1 Gb in *Machilis* (Gassner et al., 2014)). Moreover, RNA‐seq data and transcriptome assemblies often become available at an early stage of genome projects as they are a valuable resources for the functional annotation of a reference genome and can thus be leveraged to gain early insights into the population genetics of the focal organism.

Using nuclear and mitochondrial RNA‐seq data for 17 individuals from six sampling localities, we assess different types of variation – mitochondrial as well as rare and common nuclear variants – in an attempt to capture and reconstruct different aspects of the evolutionary history of *M. pallida* and reassess previous work. In particular, the suggested parthenogenetic mode of reproduction (Wachter et al., 2012) and polyploidy (Gassner et al., 2014) in *M. pallida* certainly affect our expectations regarding the genetic patterns revealed by the different kinds of variation under various evolutionary scenarios.

Population genetic theory predicts that asexual populations should (1) have lower effective population sizes (Barton & Charlesworth, 1998), and (2) be less efficient in their adaptive potential, since beneficial mutations would be lost more easily in asexual compared with sexual populations (Muller, 1964). A beneficial mutation needs to confer a strong selective advantage to avoid loss through clonal interference and Muller's Ratchet (Charlesworth & Charlesworth, 1997; Felsenstein, 1974; Fisher, 1930; Hill & Robertson, 1966; Muller, 1932, 1964). However, transiently abundant beneficial mutations that do not go to fixation might be common in asexual populations, which might experience a leapfrog effect, where the common genotype is less closely related to the immediately preceding common genotype, but more closely related to earlier genotypes (Gerrish & Lenski, 1998). In other words, two individuals sampled from different asexual populations might appear to be more closely related to each other than to individuals from their own population if there is more than one asexual lineage present therein.

The combination of asexuality and polyploidy as suggested for *M. pallida* is generally assumed to be relatively rare (Burch & Jung, 1993; Dufresne & Hebert, 1994; Otto & Whitton, 2000; White, 1973), and most obligately asexual lineages in plants and animals have evolved relatively recently (Maynard Smith, 1978). Moreover, it was found that polyploid populations more often tend to have multiple rather than single origins in plants (Soltis et al., 1993; Soltis & Soltis, 1999) and in animals (reviewed in Otto & Whitton, 2000, see also Chaplin & Hebert, 1997, Dufresne & Hebert, 1994). Such multiple origins of polyploidy are thought to arise via either a high rate of polyploidization during initial establishment of a given lineage and/or recurrent gene flow with related diploid taxa (Otto & Whitton, 2000).

Based on the assumptions stemming from the aforementioned theoretical work, we will now discuss the significance of the patterns found in the different variant types examined in this study. Notably, the analyses of the three different variant types revealed distinct patterns suggesting that they indeed capture different aspects of the evolutionary history of *M. pallida*.

The observed genetic patterns might be consistent with ice age survival of *M. pallida* on central and/or peripheral nunataks (as described in Wachter et al., 2012). The central position of the populations located at the main ridge of the Alps relative to the two southern populations in the mitochondrial haplotype network (Figure 2) could indicate dispersal from a central Alpine refugium.

Consistent with findings by Wachter et al. (2012), the mitochondrial data mostly reflect geography, with the two southern localities and populations from the Alpine main ridge representing three main groups (Figure 3, Figure A2, Table A6). Within the central Alpine populations, however, P forms a coherent, somewhat delimited cluster (Figure 3, Figure A2, Tables A5 and A6). While localities K and L are very close to each other and connected by a ridge, P is demarcated by a valley and O is farthest away and separated by a larger valley. Therefore, the P cluster may, at least partly, also mirror a geographic pattern. However, P is the locality with the highest number of samples, and we cannot exclude a sample size bias in our analyses. Genetic distances among all sampled mitochondrial haplotypes are low and thus do not provide direct support for a hybridization event between two distinct lineages.

The common nuclear variants show a more heterogeneous picture but are broadly consistent with the mitochondrial results confirming the differentiation of the geographically separated central Alpine and southern localities as well as the P cluster. We note, however, that the differentiation between North and South is less clear and more gradual (Figure 4, Figure A3, Tables A5 and A6). Moreover, we do not find support for the existence of two main nuclear clusters suggested by an earlier admixture analysis on AFLP data (Wachter et al., 2012) (discussed below).

The results from the rare nuclear variants are in stark contrast to the other two marker types. We do not find any obvious patterns mirroring populations or geographic distances with the sole exception of the P cluster in the PCoA, which may be driven by sample size (Figure 4, Figure A4).

For the interpretation of the observed patterns, we consider four simplistic scenarios that differ in whether parthenogenesis emerged before or after populations split and in the presence and absence of migration (Figure 6). Assuming a single origin of parthenogenesis in an unstructured population followed by immediate dispersal of this lineage to the current locations (Figure 6a,c), we would not expect to see clustering by geography. Later migration between nearby populations (Figure 6d) could, however, create a signature of geography and isolation by distance and, for example, explain the coherence of the central Alpine cluster. *Machilis* species are considered slow dispersers based on the fact that they lack wings and due to the high degree of endemism in the genus. However, their actual dispersal potential has not been quantified so far (Sturm & Machida, 2001, p. 61/62). A related scenario would involve a single origin of parthenogenesis followed by an extended time period allowing for range expansion and differentiation of the parthenogenetic lineage and subsequent fragmentation, yielding the current distribution pattern.

**FIGURE 5:**
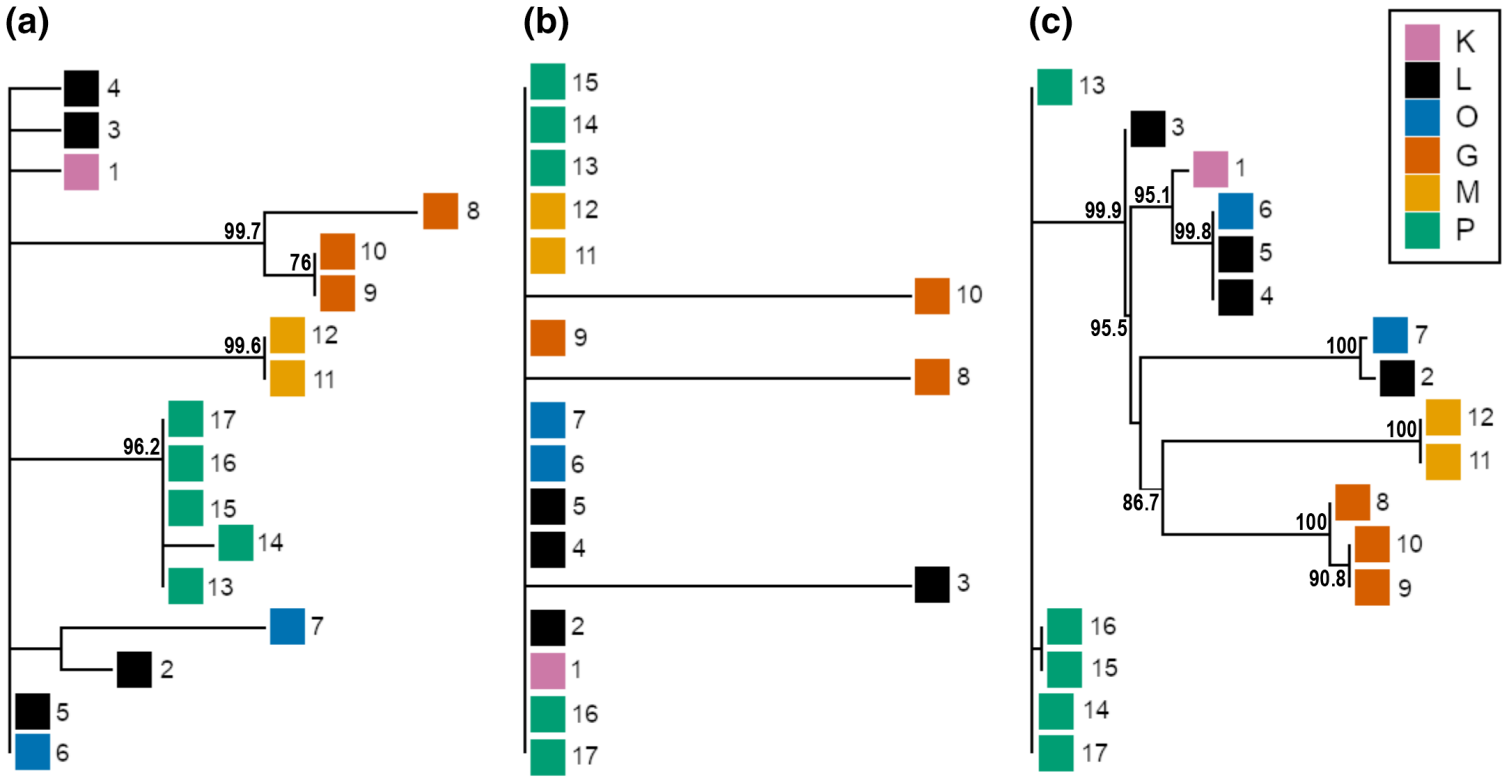
Neighbor‐joining trees for all three variant types, with (a) Mitochondrial variants (*n* = 29), (b) Rare variants (*n* = 12,126), and (c) Common variants (*n* = 201,105). Colors correspond to the different populations; Laempermahdspitze (L, black), Kesselspitze (K, pink), Padasterjochhaus (P, green), Obernberger Tribulaun (O, blue), Murmeltierhuette (M, yellow) and Grosté Seilbahn Bergstation (G, orange). Bootstrap values above 70% are shown.

However, as mentioned before, there could be different scenarios, congruent with the observed data. For instance, the neighbor‐joining tree constructed from common nuclear variants (Figure 5c) proposes a closer relationship between the two southern populations consistent with the existence of central and peripheral refugia. Additionally, the aforementioned leapfrog effect may also affect the observed relationships between populations, and thus, interpreting these patterns warrants caution.

**FIGURE 6:**
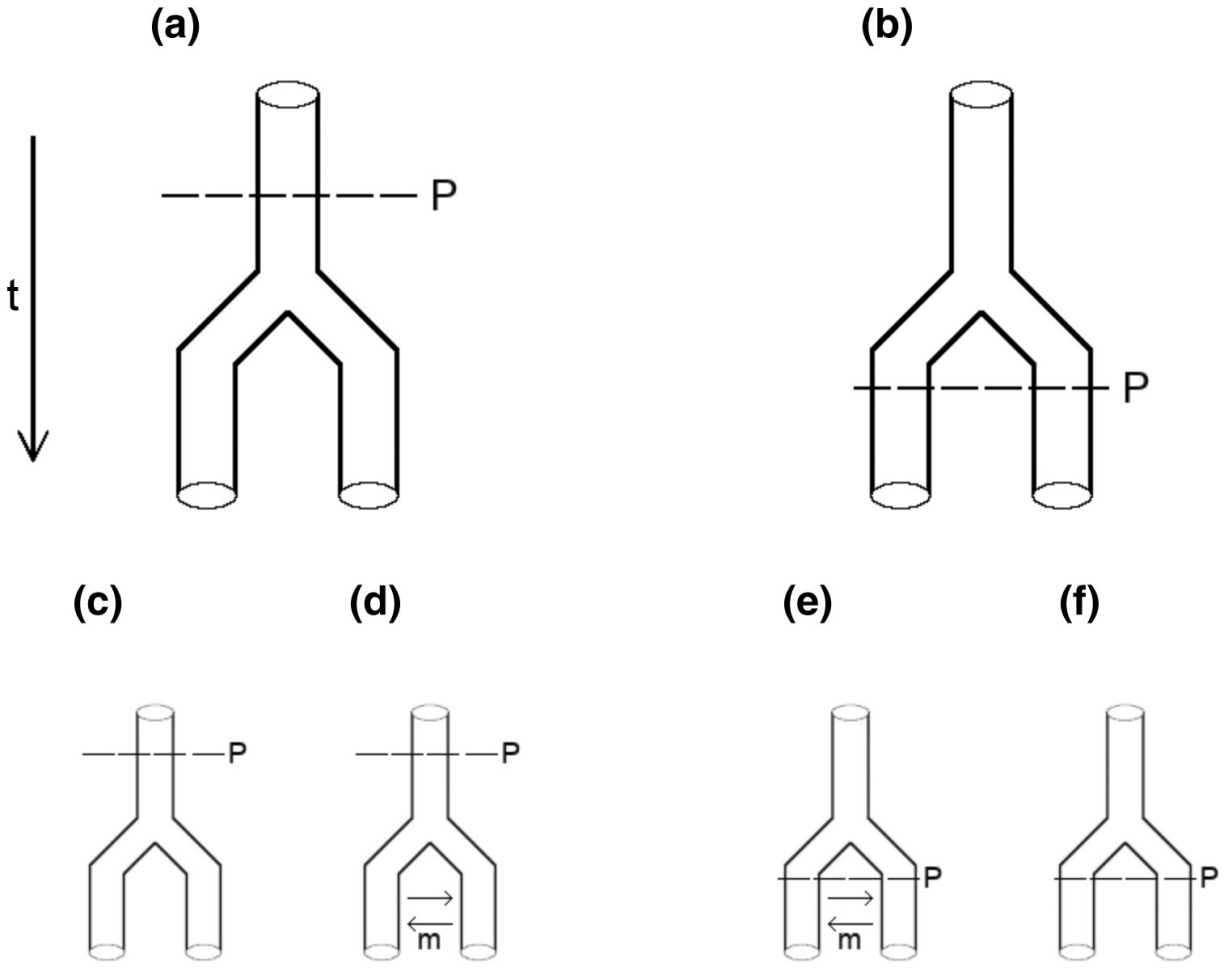
Hierarchical representation of simplified putative scenarios of the evolutionary history of *Machilis pallida* that differ in the timing of the onset of parthenogenesis (a, b) and the presence of migration (c–f).

Alternatively, the clusters visible in the mitochondrial and common nuclear data may reflect population structure already present in a more widespread sexual progenitor, whereas the lack of structure in the rare nuclear variants may be attributable to the presence of asexual lineages as well as limited resolution due to the small sample sizes for single populations. In this scenario, multiple transitions to parthenogenesis after the build‐up of population structure need to be invoked (Figure 6b,e,f) but it would not be necessary to postulate migration to explain the geographic clusters (Figure 6e,f). The fact that the mitochondrial variants show more distinct clusters compared with the common nuclear variants is consistent with the smaller effective population size of mitochondrial DNA and therefore a stronger effect of drift. Alternatively, common nuclear variants could exhibit the aforementioned leapfrog effect (Gerrish & Lenski, 1998). That is, if enough common variants show a pattern, where an individual appears more closely related to a conspecific from a different population, then this could lead to common nuclear variants exhibiting a different picture than rare nuclear or mitochondrial variants, where individuals from different populations form tight clusters. However, while common nuclear variants do in fact show such a pattern, we cannot make a definitive statement as to the underlying processes. While we focus on a few simple scenarios here, we note that arbitrarily complex scenarios including combinations of multiple, temporally separated origins of parthenogenesis, rare sexual reproduction, colonization and extinction cycles, and stepping stone models could also fit our data.

Wachter et al. (2012) proposed central and peripheral nunatak survival based on the geographic distribution of mitochondrial COI haplotypes and two nuclear AFLP clusters across three sampling localities. Under the assumption of slow and limited dispersal of this apterygote insect species, our data could be explained by such a scenario. In contrast to these previous results, however, we find no evidence for the presence of two distinct nuclear clusters but instead reveal a more complex population structure. This may be due to differences in marker types and sample size or due to the *k* = 2 conundrum (Janes et al., 2017), the tendency of the deltaK method (Evanno et al., 2005) to frequently identify *k* = 2 as top hierarchical level in STRUCTURE analyses (Pritchard et al., 2000). Moreover, our strategy of leveraging the information contained in sets of different variants highlights possible, more complex variations of this simplistic scenario as outlined above despite the small sample size. Therefore, applying this approach to a larger sampling scheme, both in terms of populations and individuals, combined with simulation studies holds great potential to fully elucidate the distribution and migration patterns during the ice ages, rigorously test competing hypotheses and date dispersal events as well as the onset of parthenogenesis.

Polyploidization is highly associated with parthenogenetic reproduction in animals and is also frequently correlated with hybridization (Otto & Whitton, 2000). It is conceivable that polyploidization events were accompanied by transitions to parthenogenesis in *M. pallida* and that hybridization was involved (e.g. Dejaco et al., 2016). Moreover, since multiple origins of polyploidy are not uncommon (reviewed in Chaplin & Hebert, 1997; Dufresne & Hebert, 1994; Otto & Whitton, 2000; Soltis et al., 1993; Soltis & Soltis, 1999), the putative joint occurrence of parthenogenesis and polyploidy would neither contradict scenarios involving a single or multiple origins of parthenogenesis. To actually assess these relationships, however, additional work is required.

## 5 CONCLUSIONS

In this study, we assess genetic patterns for different variant types in a parthenogenetic, triploid, and endemic Alpine bristletail species. We demonstrate that mitochondrial and common nuclear variants mirror geographic patterns. Moreover, we highlight that different types of variants capture different aspects of the evolutionary history of the species and outline the potential of their combined consideration for unraveling more complex scenarios. We emphasize that *M. pallida* and the genus *Machilis*, in general, represent an interesting study system for the evolution of different sexual strategies, polyploidization, and genome re‐organization, as well as for adaptation to environmental change. We also highlight the need for further studies and the presented novel resources, transcriptome, and mitochondrial assemblies together with transcriptome data for 17 individuals, will facilitate future research in this diverse system.

## AUTHOR CONTRIBUTIONS

**Marlene Haider:** Formal analysis (equal); methodology (equal); visualization (supporting); writing – original draft (supporting). **Martin P. Schilling:** Data curation (equal); formal analysis (equal); methodology (equal); software (equal); visualization (equal); writing – original draft (equal). **Markus H. Moest:** Data curation (equal); methodology (supporting); writing – original draft (equal). **Florian M. Steiner:** Conceptualization (equal); funding acquisition (equal); project administration (equal); resources (equal); supervision (equal); writing – review and editing (equal). **Birgit C. Schlick‐Steiner:** Conceptualization (equal); funding acquisition (equal); project administration (equal); resources (equal); supervision (equal); writing – review and editing (equal). **Wolfgang Arthofer:** Conceptualization (equal); methodology (equal); writing – original draft (supporting).

## FUNDING INFORMATION

This research was funded in part by the Austrian Science Fund (FWF): P30861 and the Autonomous Province of South Tyrol (project‐ID: 1/40.3; 27 January 2014).

## CONFLICT OF INTEREST STATEMENT

None.

## Data Availability

DNA sequences: European Nucleotide Archive (ENA) study accession ERP120116 Individual accessions and sample information: Table A7 Transcriptome assembly: European Nucleotide Archive (ENA) accession ERZ1673957 Transcriptome annotation and GO terms: Table A2 deposited on https://github.com/mphaider/M.pallida.git and will be deposited on Dryad. Mitochondrial assembly: European Nucleotide Archive (ENA) accession ERZ1668275 Code for de novo transcriptome assembly and variant calling: https://github.com/mphaider/M.pallida.git and will be deposited on Dryad. Code for mitochondrial variant calling and population genetics analyses: https://github.com/schimar/mpallida19.git and will be deposited on Dryad.

## APPENDIX 1

**TABLE A1:** Information on sample IDs, read numbers during read processing, coverage of reads and GC content.

ID	Individual	*N* reads raw (single)	*N* reads after quality filtering	*N* reads after decontamination	*N* reads after normalization	Coverage (mean ± SD)	GC content [%]
1	92,360	18,356,467	36,247,250	36,247,230	6,629,620	7.02 ± 19.98	40.63
2	92,361	26,141,248	51,662,040	51,662,022	9,576,382	10.14 ± 24.89	41.88
3	92,362	25,381,606	50,178,814	50,178,794	9,812,710	10.39 ± 25.58	40.6
4	92,363	28,471,747	56,414,038	56,414,014	11,374,509	12.07 ± 27.51	41.77
5	92,364	18,604,788	36,863,302	36,863,290	8,234,937	8.74 ± 23.07	42.99
6	92,365	20,013,276	39,559,548	39,559,530	8,164,247	8.68 ± 23.09	40.78
7	92,366	23,176,562	45,803,428	45,803,404	9,023,848	9.61 ± 23.81	42.71
8	92,367	20,324,156	40,180,388	40,180,372	7,418,644	7.87 ± 22.51	42.32
9	92,368	25,281,428	49,979,044	49,978,924	9,740,077	10.30 ± 25.49	41.09
10	92,369	17,042,850	33,707,928	33,707,822	6,535,554	7.00 ± 19.97	41.24
11	92,370	16,068,604	31,678,722	31,678,652	5,559,225	5.87 ± 18.49	41.12
12	92,371	17,989,323	35,660,912	35,660,836	6,805,716	7.24 ± 21.04	40.88
13	92,372	13,016,877	25,638,612	25,638,592	5,694,169	6.05 ± 18.37	40.8
14	92,373	17,648,063	34,718,940	34,718,882	7,340,238	7.74 ± 21.59	40.43
15	92,374	16,835,713	33,167,428	33,167,402	7,469,014	7.88 ± 21.43	40.3
16	92,375	21,532,793	42,572,006	42,571,970	8,901,355	9.38 ± 23.55	40.19
17	92,376	26,486,877	52,445,844	52,445,818	10,417,939	10.89 ± 26.22	39.65

**TABLE A2:** A summary of the annotation and gene ontology analysis can be found in Table A2 deposited at: https://github.com/mphaider/M.pallida.git.

**TABLE A3:** Information on the location of the 10 longest open reading frames (ORFs) on the *Machilis pallida* mitochondrium and corresponding best SmartBlast hits.

Label	Strand	Frame	Start	Stop	LenNT	LenAA	SmartBlast	Accession
ORF45	−	2	8392	6629	1764	587	NADH dehydrogenase subunit V	YP_009047273.1
ORF53	−	3	9807	8458	1350	449	NADH dehydrogenase subunit IV	YP_009047274.1
ORF25	+	3	10,758	11,894	1137	378	Cytochrome b	YP_009047277.1
ORF13	+	2	554	1588	1035	344	NADH dehydrogenase subunit II	YP_009047266.1
ORF51	−	3	12,948	11,980	969	322	NADH dehydrogenase subunit I	YP_009047278.1
ORF14	+	2	3266	4120	855	284	Cytochrome *c* Oxidase subunit II	YP_009047268.1
ORF5	+	1	5059	5886	828	275	Cytochrome *c* Oxidase subunit III	YP_009047271.1
ORF15	+	2	4382	5059	678	225	ATP synthase F0 subunit 6	YP_009047270.1
ORF20	+	3	2676	3332	657	218	Cytochrome c oxidase subunit I	YP_009047267.1
ORF50	−	2	2410	1946	465	154	Cytochrome oxidase subunit I	SSC84599.1

Abbreviations: LenAA, length in amino acids; LenNT, length in nucleotides.

**TABLE A4:** Information on location of protein and RNA coding genes on the *Machilis pallida* mitochondrion.

Protein/RNA coding genes	Start (bp)	Stop (bp)
NADH dehydrogenase subunit 1	11,992	12,906
NADH dehydrogenase subunit 2	554	1522
NADH dehydrogenase subunit 3	5908	6258
NADH dehydrogenase subunit 4	8470	9807
NADH dehydrogenase subunit 4 L	9804	10,073
NADH dehydrogenase subunit 5	6665	8305
NADH dehydrogenase subunit 6	10,255	10,749
atp6	4385	5050
atp8	4227	4385
cob	10,758	11,873
cox1	1788	3308
cox2	3398	4075
cox3	5065	5835
rrnS	14,404	15,222
rrnL	12,946	14,326

**TABLE A5:** Pairwise distance matrix between individuals using Nei's distance. Genetic distances using nuclear not fixed variants (below the diagonal) and mitochondrial variants (above diagonal).

	1 (K)	2 (L)	3 (L)	4 (L)	5 (L)	6 (O)	7 (O)	8 (G)	9 (G)	10 (G)	11 (M)	12 (M)	13 (P)	14 (P)	15 (P)	16 (P)	17 (P)
1 (K)		0.12	0.071	0.071	0.035	0.035	0.245	0.372	0.276	0.276	0.232	0.232	0.16	0.189	0.148	0.148	0.148
2 (L)	0.075		0.12	0.12	0.081	0.081	0.193	0.441	0.338	0.338	0.291	0.291	0.214	0.245	0.202	0.202	0.202
3 (L)	0.072	0.064		0.071	0.035	0.035	0.245	0.372	0.276	0.276	0.232	0.232	0.16	0.189	0.148	0.148	0.148
4 (L)	0.07	0.062	0.059		0.035	0.035	0.245	0.372	0.276	0.276	0.232	0.232	0.16	0.189	0.148	0.148	0.148
5 (L)	0.079	0.072	0.069	0.064		0	0.202	0.323	0.232	0.232	0.189	0.189	0.12	0.148	0.109	0.109	0.109
6 (O)	0.078	0.071	0.068	0.063	0.073		0.202	0.323	0.232	0.232	0.189	0.189	0.12	0.148	0.109	0.109	0.109
7 (O)	0.077	0.064	0.066	0.064	0.073	0.073		0.618	0.496	0.496	0.441	0.441	0.354	0.388	0.338	0.338	0.338
8 (G)	0.082	0.075	0.073	0.07	0.079	0.078	0.077		0.148	0.148	0.595	0.595	0.496	0.534	0.477	0.477	0.477
9 (G)	0.076	0.069	0.066	0.066	0.074	0.074	0.071	0.071		0	0.477	0.477	0.388	0.423	0.372	0.372	0.372
10 (G)	0.09	0.084	0.082	0.082	0.088	0.089	0.086	0.086	0.08		0.477	0.477	0.388	0.423	0.372	0.372	0.372
11 (M)	0.093	0.084	0.082	0.081	0.089	0.088	0.085	0.089	0.084	0.098		0	0.338	0.372	0.323	0.323	0.323
12 (M)	0.085	0.074	0.073	0.071	0.08	0.079	0.075	0.08	0.075	0.089	0.086		0.338	0.372	0.323	0.323	0.323
13 (P)	0.077	0.068	0.065	0.063	0.073	0.072	0.069	0.076	0.069	0.085	0.087	0.077		0.044	0.009	0.009	0.009
14 (P)	0.082	0.072	0.07	0.067	0.077	0.076	0.073	0.079	0.073	0.089	0.091	0.082	0.069		0.035	0.035	0.035
15 (P)	0.08	0.072	0.069	0.067	0.077	0.075	0.074	0.079	0.073	0.088	0.088	0.079	0.068	0.073		0	0
16 (P)	0.076	0.066	0.063	0.061	0.072	0.07	0.068	0.074	0.067	0.084	0.086	0.077	0.063	0.067	0.064		0
17 (P)	0.076	0.066	0.063	0.061	0.072	0.07	0.068	0.074	0.067	0.083	0.086	0.077	0.062	0.067	0.063	0.057	

**TABLE A6:** Pairwise distance matrix between populations using Nei's distance. Genetic distances using nuclear not fixed variants (below the diagonal) and mitochondrial variants (above diagonal).

	K	L	O	G	M	P
K		0.046	0.086	0.275	0.232	0.152
L	0.05		0.048	0.251	0.207	0.126
O	0.06	0.027		0.309	0.259	0.173
G	0.057	0.03	0.039		0.483	0.381
M	0.069	0.035	0.044	0.04		0.328
P	0.053	0.019	0.028	0.026	0.038	

**TABLE A7:** European Nucleotide Archive (ENA) accessions for samples and raw reads sequenced in this study.

Primary accession	Secondary accession	Unique name	Study	Experiment
ERS4357532	SAMEA6593248	92,010	ERP120116	ERX4639242
ERS4357531	SAMEA6593247	92,376	ERP120116	ERX4639621
ERS4357530	SAMEA6593246	92,375	ERP120116	ERX4639620
ERS4357529	SAMEA6593245	92,374	ERP120116	ERX4639619
ERS4357528	SAMEA6593244	92,373	ERP120116	ERX4639618
ERS4357527	SAMEA6593243	92,372	ERP120116	ERX4639617
ERS4357526	SAMEA6593242	92,371	ERP120116	ERX4639616
ERS4357525	SAMEA6593241	92,370	ERP120116	ERX4639615
ERS4357524	SAMEA6593240	92,369	ERP120116	ERX4639614
ERS4357523	SAMEA6593239	92,368	ERP120116	ERX4639613
ERS4357522	SAMEA6593238	92,367	ERP120116	ERX4639612
ERS4357521	SAMEA6593237	92,366	ERP120116	ERX4639611
ERS4357520	SAMEA6593236	92,365	ERP120116	ERX4639610
ERS4357519	SAMEA6593235	92,364	ERP120116	ERX4639609
ERS4357518	SAMEA6593234	92,363	ERP120116	ERX4639606
ERS4357517	SAMEA6593233	92,362	ERP120116	ERX4639600
ERS4357516	SAMEA6593232	92,361	ERP120116	ERX4639597
ERS4357515	SAMEA6593231	92,360	ERP120116	ERX4639565
ERS5328265	SAMEA7571643	Combined_92,360–92,376	ERP120116	ERX4706073

## References

[ece310227-bib-0001] Abbott, R. J. , & Brochmann, C. (2003). History and evolution of the arctic flora: In the footsteps of Eric Hultén. Molecular Ecology, 12, 299–313. 12535083 10.1046/j.1365-294x.2003.01731.x

[ece310227-bib-0002] Altschul, S. F. , Gish, W. , Miller, W. , Myers, E. W. , & Lipman, D. J. (1990). Basic local alignment search tool. Journal of Molecular Biology, 215, 403–410. 2231712 10.1016/S0022-2836(05)80360-2

[ece310227-bib-0003] Andrews, S. (2010). FastQC: A quality control tool for high throughput sequence data . https://www.bioinformatics.babraham.ac.uk/projects/fastqc/

[ece310227-bib-0004] Ashburner, M. , Ball, C. A. , Blake, J. A. , Botstein, D. , Butler, H. , Cherry, J. M. , Davis, A. P. , Dolinski, K. , Dwight, S. S. , Eppig, J. T. , Harris, M. A. , Hill, D. P. , Issel‐Tarver, L. , Kasarskis, A. , Lewis, S. , Matese, J. C. , Richardson, J. E. , Ringwald, M. , Rubin, G. M. , & Sherlock, G. (2000). Gene ontology: Tool for the unification of biology. Nature Genetics, 25, 25–29. 10802651 10.1038/75556 PMC3037419

[ece310227-bib-0005] Avise, J. C. , Arnold, J. , Ball, R. M. , Bermingham, E. , Lamb, T. , Neigel, J. E. , Reeb, C. A. , & Saunders, N. C. (1987). Intraspecific phylogeography: The mitochondrial DNA bridge between population genetics and systematics. Annual Review of Ecology and Systematics, 18, 489–522.

[ece310227-bib-0006] Baldo, L. , de Queiroz, A. , Hedin, M. , Hayashi, C. Y. , & Gatesy, J. (2011). Nuclear–mitochondrial sequences as witnesses of past interbreeding and population diversity in the jumping bristletail *Mesomachilis* . Molecular Biology and Evolution, 28, 195–210. 20667982 10.1093/molbev/msq193

[ece310227-bib-0007] Barton, N. H. , & Charlesworth, B. (1998). Why sex and recombination? Science, 281, 1986–1990. 9748151

[ece310227-bib-0008] Barton, N. H. , & Slatkin, M. (1986). A quasi‐equilibrium theory of the distribution of rare alleles in a subdivided population. Heredity, 56, 409–415. 3733460 10.1038/hdy.1986.63

[ece310227-bib-0009] Bast, J. , Parker, D. J. , Dumas, Z. , Jalvingh, K. M. , Tran Van, P. , Jaron, K. S. , Figuet, E. , Brandt, A. , Galtier, N. , & Schwander, T. (2018). Consequences of asexuality in natural populations: Insights from stick insects. Molecular Biology and Evolution, 35, 1668–1677. 29659991 10.1093/molbev/msy058 PMC5995167

[ece310227-bib-0010] Bernt, M. , Donath, A. , Jühling, F. , Externbrink, F. , Florentz, C. , Fritzsch, G. , Pütz, J. , Middendorf, M. , & Stadler, P. F. (2013). MITOS: Improved de novo metazoan mitochondrial genome annotation. Molecular Phylogenetics and Evolution, 69, 313–319. 22982435 10.1016/j.ympev.2012.08.023

[ece310227-bib-0011] Bettin, O. , Cornejo, C. , Edwards, P. J. , & Holderegger, R. (2007). Phylogeography of the high alpine plant *Senecio halleri* (Asteraceae) in the European Alps: In situ glacial survival with postglacial stepwise dispersal into peripheral areas. Molecular Ecology, 16, 2517–2524. 17561910 10.1111/j.1365-294X.2007.03273.x

[ece310227-bib-0012] Birky, C. W. (1996). Heterozygosity, heteromorphy, and phylogenetic trees in asexual eukaryotes. Genetics, 144, 427–437. 8878706 10.1093/genetics/144.1.427 PMC1207515

[ece310227-bib-0013] Brandt, A. , Schaefer, I. , Glanz, J. , Schwander, T. , Maraun, M. , Scheu, S. , & Bast, J. (2017). Effective purifying selection in ancient asexual oribatid mites. Nature Communications, 8, 873. 10.1038/s41467-017-01002-8 PMC5638860 29026136

[ece310227-bib-0014] Broad Institute . (2018). Picard tools. Broad Institute. https://broadinstitute.github.io/picard/

[ece310227-bib-0015] Bryant, D. M. , Johnson, K. , DiTommaso, T. , Tickle, T. , Couger, M. B. , Payzin‐Dogru, D. , Lee, T. J. , Leigh, N. D. , Kuo, T.‐H. , Davis, F. G. , Bateman, J. , Bryant, S. , Guzikowski, A. R. , Tsai, S. L. , Coyne, S. , Ye, W. W. , Freeman, R. M. , Peshkin, L. , Tabin, C. J. , … Whited, J. L. (2017). A tissue‐mapped axolotl *de novo* transcriptome enables identification of limb regeneration factors. Cell Reports, 18, 762–776. 28099853 10.1016/j.celrep.2016.12.063 PMC5419050

[ece310227-bib-0016] Burch, J. B. , & Jung, Y. (1993). Polyploid chromosome numbers in the *Torquis* group of the freshwater snail genus *Gyraulus* (Mollusca: Pulmonata: Planorbidae). Cytologia, 58, 145–149.

[ece310227-bib-0017] Bushnell, B. (2021). BBMap . https://sourceforge.net/projects/bbmap/

[ece310227-bib-0018] Chaplin, J. A. , & Hebert, P. D. N. (1997). *Cyprinotus incongruens* (Ostracoda): An ancient asexual? Molecular Ecology, 6, 155–168.

[ece310227-bib-0019] Charlesworth, B. , & Charlesworth, D. (1997). Rapid fixation of deleterious alleles can be caused by Muller's ratchet. Genetical Research, 70, 63–73. 9369098 10.1017/s0016672397002899

[ece310227-bib-0020] Chevreux, B. , Wetter, T. , & Suhai, S. (1999). Genome sequence assembly using trace signals and additional sequence information. German Conference on Bioinformatics, 99, 45–56.

[ece310227-bib-0021] David, K. T. (2022). Global gradients in the distribution of animal polyploids. Proceedings of the National Academy of Sciences of the United States of America, 119, e2214070119. 36409908 10.1073/pnas.2214070119 PMC9860298

[ece310227-bib-0022] de Jong, Y. , Verbeek, M. , Michelsen, V. , Bjørn, P. D. P. , Los, W. , Steeman, F. , Bailly, N. , Basire, C. , Chylarecki, P. , Stloukal, E. , Hagedorn, G. , Wetzel, F. , Glöckler, F. , Kroupa, A. , Korb, G. , Hoffmann, A. , Häuser, C. , Kohlbecker, A. , Müller, A. , … Penev, L. (2014). Fauna Europaea – All European animal species on the web. Biodiversity Data Journal, 2, e4034. 10.3897/BDJ.2.e4034 PMC4206781 25349527

[ece310227-bib-0023] Dejaco, T. , Arthofer, W. , Sheets, H. D. , Moder, K. , Thaler‐Knoflach, B. , Christian, E. , Mendes, L. F. , Schlick‐Steiner, B. C. , & Steiner, F. M. (2012). A toolbox for integrative species delimitation in *Machilis* jumping bristletails (Microcoryphia: Machilidae). Zoologischer Anzeiger, 251, 307–316.

[ece310227-bib-0024] Dejaco, T. , Gassner, M. , Arthofer, W. , Schlick‐Steiner, B. C. , & Steiner, F. M. (2016). Taxonomist's nightmare … evolutionist's delight: An integrative approach resolves species limits in jumping bristletails despite widespread hybridization and parthenogenesis. Systematic Biology, 65, 947–974. 26869489 10.1093/sysbio/syw003 PMC5066060

[ece310227-bib-0025] Dufresne, F. , & Hebert, P. D. N. (1994). Hybridization and origins of polyploidy. Proceedings of the Royal Society B: Biological Sciences, 258, 141–146.

[ece310227-bib-0026] Ellegren, H. , & Galtier, N. (2016). Determinants of genetic diversity. Nature Reviews. Genetics, 17, 422–433. 10.1038/nrg.2016.58 27265362

[ece310227-bib-0027] Evanno, G. , Regnaut, S. , & Goudet, J. (2005). Detecting the number of clusters of individuals using the software STRUCTURE: A simulation study. Molecular Ecology, 14, 2611–2620. 15969739 10.1111/j.1365-294X.2005.02553.x

[ece310227-bib-0028] Felsenstein, J. (1974). The evolutionary advantage of recombination. Genetics, 78, 737–756. 4448362 10.1093/genetics/78.2.737 PMC1213231

[ece310227-bib-0029] Feng, S. , Wan, W. , Li, Y. , Wang, D. , Ren, G. , Ma, T. , & Ru, D. (2023). Transcriptome‐based analyses of adaptive divergence between two closely related spruce species on the Qinghai–Tibet plateau and adjacent regions. Molecular Ecology, 32, 476–491. 36320185 10.1111/mec.16758

[ece310227-bib-0030] Finn, R. D. , Bateman, A. , Clements, J. , Coggill, P. , Eberhardt, R. Y. , Eddy, S. R. , Heger, A. , Hetherington, K. , Holm, L. , Mistry, J. , Sonnhammer, E. L. L. , Tate, J. , & Punta, M. (2014). Pfam: The protein families database. Nucleic Acids Research, 42, D222–D230. 24288371 10.1093/nar/gkt1223 PMC3965110

[ece310227-bib-0031] Finn, R. D. , Clements, J. , & Eddy, S. R. (2011). HMMER web server: Interactive sequence similarity searching. Nucleic Acids Research, 39, W29–W37. 21593126 10.1093/nar/gkr367 PMC3125773

[ece310227-bib-0032] Fisher, R. A. (1930). The genetical theory of natural selection. The Clarendon Press.

[ece310227-bib-0033] Fu, L. , Niu, B. , Zhu, Z. , Wu, S. , & Li, W. (2012). CD‐HIT: Accelerated for clustering the next‐generation sequencing data. Bioinformatics, 28, 3150–3152. 23060610 10.1093/bioinformatics/bts565 PMC3516142

[ece310227-bib-0034] Funk, D. J. , & Omland, K. E. (2003). Species‐level paraphyly and polyphyly: Frequency, causes, and consequences, with insights from animal mitochondrial DNA. Annual Review of Ecology, Evolution, and Systematics, 34, 397–423.

[ece310227-bib-0035] Gassner, M. , Dejaco, T. , Schönswetter, P. , Marec, F. , Arthofer, W. , Schlick‐Steiner, B. C. , & Steiner, F. M. (2014). Extensive variation in chromosome number and genome size in sexual and parthenogenetic species of the jumping‐bristletail genus *Machilis* (Archaeognatha). Ecology and Evolution, 4, 4093–4105. 25505536 10.1002/ece3.1264 PMC4242562

[ece310227-bib-0036] Gerrish, P. J. , & Lenski, R. E. (1998). The fate of competing beneficial mutations in an asexual population. Genetica, 102, 127. 9720276

[ece310227-bib-0037] Gompert, Z. , Lucas, L. K. , Buerkle, C. A. , Forister, M. L. , Fordyce, J. A. , & Nice, C. C. (2014). Admixture and the organization of genetic diversity in a butterfly species complex revealed through common and rare genetic variants. Molecular Ecology, 23(4555), 4573. 10.1111/mec.12811 24866941

[ece310227-bib-0038] Grabherr, M. G. , Haas, B. J. , Yassour, M. , Levin, J. Z. , Thompson, D. A. , Amit, I. , Adiconis, X. , Fan, L. , Raychowdhury, R. , Zeng, Q. , Chen, Z. , Mauceli, E. , Hacohen, N. , Gnirke, A. , Rhind, N. , di Palma, F. , Birren, B. W. , Nusbaum, C. , Lindblad‐Toh, K. , … Regev, A. (2011). Full‐length transcriptome assembly from RNA‐Seq data without a reference genome. Nature Biotechnology, 29, 644–652. 10.1038/nbt.1883 PMC3571712 21572440

[ece310227-bib-0039] Haas, B. J. , Papanicolaou, A. , Yassour, M. , Grabherr, M. , Blood, P. D. , Bowden, J. , Couger, M. B. , Eccles, D. , Li, B. , Lieber, M. , MacManes, M. D. , Ott, M. , Orvis, J. , Pochet, N. , Strozzi, F. , Weeks, N. , Westerman, R. , William, T. , Dewey, C. N. , … Regev, A. (2013). *De novo* transcript sequence reconstruction from RNA‐seq using the Trinity platform for reference generation and analysis. Nature Protocols, 8, 1494–1512. 23845962 10.1038/nprot.2013.084 PMC3875132

[ece310227-bib-0040] Hahn, C. , Bachmann, L. , & Chevreux, B. (2013). Reconstructing mitochondrial genomes directly from genomic nextgeneration sequencing reads—A baiting and iterative mapping approach. Nucleic Acids Research, 41, e129. 23661685 10.1093/nar/gkt371 PMC3711436

[ece310227-bib-0041] Harrison, R. G. (1989). Animal mitochondrial DNA as a genetic marker in population and evolutionary biology. Trends in Ecology & Evolution, 4, 6–11. 21227301 10.1016/0169-5347(89)90006-2

[ece310227-bib-0042] He, K. , Zhang, J.‐Y. , Deng, K.‐Z. , & Chen, Z. (2013). The complete mitochondrial genome of the bristletail *Songmachilis xinxiangensis* (Archaeognatha: Machilidae). Mitochondrial DNA, 24, 99–101. 23005251 10.3109/19401736.2012.723001

[ece310227-bib-0043] Hill, W. G. , & Robertson, A. (1966). The effect of linkage on limits to artificial selection. Genetical Research, 8, 269–294. 5980116

[ece310227-bib-0044] Hirsch, C. D. , Evans, J. , Buell, C. R. , & Hirsch, C. N. (2014). Reduced representation approaches to interrogate genome diversity in large repetitive plant genomes. Briefings in Functional Genomics, 13, 257–267. 24395692 10.1093/bfgp/elt051

[ece310227-bib-0045] Holderegger, R. , & Thiel‐Egenter, C. (2009). A discussion of different types of glacial refugia used in mountain biogeography and phylogeography. Journal of Biogeography, 36, 476–480.

[ece310227-bib-0046] Huerta‐Cepas, J. , Szklarczyk, D. , Forslund, K. , Cook, H. , Heller, D. , Walter, M. C. , Rattei, T. , Mende, D. R. , Sunagawa, S. , Kuhn, M. , Jensen, L. J. , von Mering, C. , & Bork, P. (2016). eggNOG 4.5: A hierarchical orthology framework with improved functional annotations for eukaryotic, prokaryotic and viral sequences. Nucleic Acids Research, 44, D286–D293. 26582926 10.1093/nar/gkv1248 PMC4702882

[ece310227-bib-0047] Janes, J. K. , Miller, J. M. , Dupuis, J. R. , Malenfant, R. M. , Gorrell, J. C. , Cullingham, C. I. , & Andrew, R. L. (2017). The *K* = 2 conundrum. Molecular Ecology, 26, 3594–3602. 28544181 10.1111/mec.14187

[ece310227-bib-0048] Janetschek, H. (1949). Beitrag zur Kenntnis der Felsenspringer (Thysanura, Machilidae) Nordtirols. Veröffentlichungen des Museums Ferdinandeum (Innsbruck), 26/29, 147–165.

[ece310227-bib-0049] Janetschek, H. (1956). Das Problem der inneralpinen Eiszeitüberdauerung durch Tiere (Ein Beitrag zur Geschichte der Nivalfauna). Österreichische Zoologische Zeitschrift, 6, 421–506.

[ece310227-bib-0050] Jaron, K. S. , Bast, J. , Nowell, R. W. , Ranallo‐Benavidez, T. R. , Robinson‐Rechavi, M. , & Schwander, T. (2021). Genomic features of parthenogenetic animals. The Journal of Heredity, 112, 19–33. 32985658 10.1093/jhered/esaa031 PMC7953838

[ece310227-bib-0051] Jaron, K. S. , Parker, D. J. , Anselmetti, Y. , Tran Van, P. , Bast, J. , Dumas, Z. , Figuet, E. , François, C. M. , Hayward, K. , Rossier, V. , Simion, P. , Robinson‐Rechavi, M. , Galtier, N. , & Schwander, T. (2022). Convergent consequences of parthenogenesis on stick insect genomes. Science Advances, 8, eabg3842. 35196080 10.1126/sciadv.abg3842 PMC8865771

[ece310227-bib-0052] Kanehisa, M. , Goto, S. , Sato, Y. , Furumichi, M. , & Tanabe, M. (2012). KEGG for integration and interpretation of large‐scale molecular data sets. Nucleic Acids Research, 40, D109–D114. 22080510 10.1093/nar/gkr988 PMC3245020

[ece310227-bib-0053] Knowles, L. L. (2000). Tests of Pleistocene speciation in montane grasshoppers (genus *Melanoplus*) from the Sky Islands of Western North America. Evolution, 54, 1337–1348. 11005300 10.1111/j.0014-3820.2000.tb00566.x

[ece310227-bib-0054] Krogh, A. , Larsson, B. , von Heijne, G. , & Sonnhammer, E. L. L. (2001). Predicting transmembrane protein topology with a hidden Markov model: Application to complete genomes. Journal of Molecular Biology, 305, 567–580. 11152613 10.1006/jmbi.2000.4315

[ece310227-bib-0055] Kumar, S. , Jones, M. , Koutsovoulos, G. , Clarke, M. , & Blaxter, M. (2013). Blobology: Exploring raw genome data for contaminants, symbionts and parasites using taxon‐annotated GC‐coverage plots. Frontiers in Genetics, 4, 237. 24348509 10.3389/fgene.2013.00237 PMC3843372

[ece310227-bib-0056] Langmead, B. , & Salzberg, S. L. (2012). Fast gapped‐read alignment with Bowtie 2. Nature Methods, 9, 357–359. 22388286 10.1038/nmeth.1923 PMC3322381

[ece310227-bib-0057] Li, B. , & Dewey, C. N. (2011). RSEM: Accurate transcript quantification from RNA‐Seq data with or without a reference genome. BMC Bioinformatics, 12, 323. 21816040 10.1186/1471-2105-12-323 PMC3163565

[ece310227-bib-0058] Li, H. , Handsaker, B. , Wysoker, A. , Fennell, T. , Ruan, J. , Homer, N. , Marth, G. , Abecasis, G. , Durbin, R. , & 1000 Genome Project Data Processing Subgroup . (2009). The sequence alignment/map format and SAMtools. Bioinformatics, 25, 2078–2079. 19505943 10.1093/bioinformatics/btp352 PMC2723002

[ece310227-bib-0059] Li, Y. , Vinckenbosch, N. , Tian, G. , Huerta‐Sanchez, E. , Jiang, T. , Jiang, H. , Albrechtsen, A. , Andersen, G. , Cao, H. , Korneliussen, T. , Grarup, N. , Guo, Y. , Hellman, I. , Jin, X. , Li, Q. , Liu, J. , Liu, X. , Sparsø, T. , Tang, M. , … Wang, J. (2010). Resequencing of 200 human exomes identifies an excess of low‐frequency non‐synonymous coding variants. Nature Genetics, 42, 969–972. 20890277 10.1038/ng.680

[ece310227-bib-0060] Liegeois, M. , Sartori, M. , & Schwander, T. (2020). Extremely widespread parthenogenesis and a trade‐off between alternative forms of reproduction in mayflies (Ephemeroptera). Journal of Heredity, 112, 45–57. 10.1093/jhered/esaa027 PMC7953839 32918457

[ece310227-bib-0061] Lohse, K. , Nicholls, J. A. , & Stone, G. N. (2011). Inferring the colonization of a mountain range—Refugia vs. nunatak survival in high alpine ground beetles. Molecular Ecology, 20, 394–408. 21073591 10.1111/j.1365-294X.2010.04929.x

[ece310227-bib-0062] Lorch, S. , Zeuss, D. , Brandl, R. , & Brändle, M. (2016). Chromosome numbers in three species groups of freshwater flatworms increase with increasing latitude. Ecology and Evolution, 6, 1420–1429. 27087923 10.1002/ece3.1969 PMC4775536

[ece310227-bib-0063] Magro, A. , Lecompte, E. , Hemptinne, J.‐L. , Soares, A. O. , Dutrillaux, A.‐M. , Murienne, J. , Fürsch, H. , & Dutrillaux, B. (2020). First case of parthenogenesis in ladybirds (Coleoptera: Coccinellidae) suggests new mechanisms for the evolution of asexual reproduction. Journal of Zoological Systematics and Evolutionary Research, 58, 194–208.

[ece310227-bib-0064] Mathieson, I. , & McVean, G. (2012). Differential confounding of rare and common variants in spatially structured populations. Nature Genetics, 44, 243–246. 22306651 10.1038/ng.1074 PMC3303124

[ece310227-bib-0065] Mathieson, I. , & McVean, G. (2014). Demography and the age of rare variants. PLoS Genetics, 10, e1004528. 25101869 10.1371/journal.pgen.1004528 PMC4125085

[ece310227-bib-0066] Maynard Smith, J. M. (1978). The evolution of sex. Cambridge University Press.

[ece310227-bib-0067] McKenna, A. , Hanna, M. , Banks, E. , Sivachenko, A. , Cibulskis, K. , Kernytsky, A. , Garimella, K. , Altshuler, D. , Gabriel, S. , Daly, M. , & DePristo, M. A. (2010). The genome analysis toolkit: A mapreduce framework for analyzing next‐generation DNA sequencing data. Genome Research, 20, 1297–1303. 20644199 10.1101/gr.107524.110 PMC2928508

[ece310227-bib-0068] Memon, S. , Jia, X. , Gu, L. , & Zhang, X. (2016). Genomic variations and distinct evolutionary rate of rare alleles in *Arabidopsis thaliana* . BMC Evolutionary Biology, 16, 25. 26817829 10.1186/s12862-016-0590-7 PMC4728917

[ece310227-bib-0069] Mossion, V. , Dauphin, B. , Grant, J. , Kessler, M. , Zemp, N. , & Croll, D. (2022). Transcriptome‐wide SNPs for *Botrychium lunaria* ferns enable fine‐grained analysis of ploidy and population structure. Molecular Ecology Resources, 22, 254–271. 34310066 10.1111/1755-0998.13478 PMC9291227

[ece310227-bib-0070] Muller, H. J. (1932). Some genetic aspects of sex. American Naturalist, 66, 118–138.

[ece310227-bib-0071] Muller, H. J. (1964). The relation of recombination to mutational advance. Mutation Research ‐ Fundamental and Molecular Mechanisms of Mutagenesis, 1, 2–9. 10.1016/0027-5107(64)90047-8 14195748

[ece310227-bib-0072] NCBI . (2021). NCBI smartBLAST . https://blast.ncbi.nlm.nih.gov/smartblast/

[ece310227-bib-0073] Nelson, M. R. , Wegmann, D. , Ehm, M. G. , Kessner, D. , Jean, P. S. , Verzilli, C. , Shen, J. , Tang, Z. , Bacanu, S.‐A. , Fraser, D. , Warren, L. , Aponte, J. , Zawistowski, M. , Liu, X. , Zhang, H. , Zhang, Y. , Li, J. , Li, Y. , Li, L. , … Mooser, V. (2012). An abundance of rare functional variants in 202 drug target genes sequenced in 14,002 people. Science, 337, 100–104. 22604722 10.1126/science.1217876 PMC4319976

[ece310227-bib-0074] Okonechnikov, K. , Conesa, A. , & García‐Alcalde, F. (2016). Qualimap 2: Advanced multi‐sample quality control for highthroughput sequencing data. Bioinformatics, 32, 292–294. 26428292 10.1093/bioinformatics/btv566 PMC4708105

[ece310227-bib-0075] Otto, S. P. , & Whitton, J. (2000). Polyploid incidence and evolution. Annual Review of Genetics, 34, 401–437. 10.1146/annurev.genet.34.1.401 11092833

[ece310227-bib-0076] Palissa, A. (1964). Die Tierwelt Mitteleuropas. Insekten 1. Teil. Aperygota, vol. IV. Quelle & Meyer.

[ece310227-bib-0077] Paradis, E. (2010). Pegas: An R package for population genetics with an integrated–modular approach. Bioinformatics, 26, 419–420. 20080509 10.1093/bioinformatics/btp696

[ece310227-bib-0078] Paradis, E. , & Schliep, K. (2019). Ape 5.0: An environment for modern phylogenetics and evolutionary analyses in R. Bioinformatics, 35, 526–528. 30016406 10.1093/bioinformatics/bty633

[ece310227-bib-0079] Parisod, C. , & Besnard, G. (2007). Glacial in situ survival in the Western Alps and polytopic autopolyploidy in *Biscutella laevigata* L. (Brassicaceae). Molecular Ecology, 16, 2755–2767. 17594445 10.1111/j.1365-294X.2007.03315.x

[ece310227-bib-0080] Pembleton, L. W. , Cogan, N. O. I. , & Forster, J. W. (2013). StAMPP: An R package for calculation of genetic differentiation and structure of mixed‐ploidy level populations. Molecular Ecology Resources, 13, 946–952. 23738873 10.1111/1755-0998.12129

[ece310227-bib-0081] Petersen, T. N. , Brunak, S. , von Heijne, G. , & Nielsen, H. (2011). SignalP 4.0: Discriminating signal peptides from transmembrane regions. Nature Methods, 8, 785–786. 21959131 10.1038/nmeth.1701

[ece310227-bib-0082] Pritchard, J. K. , Stephens, M. , & Donnelly, P. (2000). Inference of population structure using multilocus genotype data. Genetics, 155, 945–959. 10835412 10.1093/genetics/155.2.945 PMC1461096

[ece310227-bib-0083] R Core Team . (2020). R: A language and environment for statistical computing. R Foundation for Statistical Computing. https://www.R‐project.org/

[ece310227-bib-0084] Rinnhofer, L. J. , Roura‐Pascual, N. , Arthofer, W. , Dejaco, T. , Thaler‐Knoflach, B. , Wachter, G. A. , Christian, E. , Steiner, F. M. , & Schlick‐Steiner, B. C. (2012). Iterative species distribution modelling and ground validation in endemism research: An alpine jumping bristletail example. Biodiversity and Conservation, 21, 2845–2863.

[ece310227-bib-0085] Schilling, M. P. , Gompert, Z. , & Wolf, P. G. (in prep.). Genetic diversity and population structure of rare and vs. common alleles in the montane endemic *Boechera lasiocarpa* .

[ece310227-bib-0086] Schneeweiss, G. M. , & Schönswetter, P. (2011). A re‐appraisal of nunatak survival in arctic‐alpine phylogeography. Molecular Ecology, 20, 190–192. 21265053 10.1111/j.1365-294x.2010.04927.x

[ece310227-bib-0087] Schönswetter, P. , Stehlik, I. , Holderegger, R. , & Tribsch, A. (2005). Molecular evidence for glacial refugia of mountain plants in the European Alps. Molecular Ecology, 14, 3547–3555. 16156822 10.1111/j.1365-294X.2005.02683.x

[ece310227-bib-0088] Schönswetter, P. , Tribsch, A. , Barfuss, M. , & Niklfeld, H. (2002). Several Pleistocene refugia detected in the high alpine plant *Phyteuma globulariifolium* Sternb. & Hoppe (Campanulaceae) in the European Alps. Molecular Ecology, 11, 2637–2647. 12453246 10.1046/j.1365-294x.2002.01651.x

[ece310227-bib-0089] Simão, F. A. , Waterhouse, R. M. , Ioannidis, P. , Kriventseva, E. V. , & Zdobnov, E. M. (2015). BUSCO: Assessing genome assembly and annotation completeness with single‐copy orthologs. Bioinformatics, 31, 3210–3212. 26059717 10.1093/bioinformatics/btv351

[ece310227-bib-0090] Slatkin, M. (1985). Rare alleles as indicators of gene flow. Evolution, 39, 53–65. 28563643 10.1111/j.1558-5646.1985.tb04079.x

[ece310227-bib-0091] Slatkin, M. , & Takahata, N. (1985). The average frequency of private alleles in a partially isolated population. Theoretical Population Biology, 28, 314–331. 10.1016/0040-5809(86)90032-8 3787501

[ece310227-bib-0092] Soltis, D. E. , & Soltis, P. S. (1999). Polyploidy: Recurrent formation and genome evolution. Trends in Ecology & Evolution, 14, 348–352. 10441308 10.1016/s0169-5347(99)01638-9

[ece310227-bib-0093] Soltis, D. E. , Soltis, P. S. , & Rieseberg, D. L. H. (1993). Molecular data and the dynamic nature of polyploidy. Critical Reviews in Plant Sciences, 12, 243–273.

[ece310227-bib-0094] Stehlik, I. (2003). Resistance or emigration? Response of alpine plants to the ice ages. Taxon, 52, 499–510.

[ece310227-bib-0095] Stehlik, I. , Blattner, F. R. , Holderegger, R. , & Bachmann, K. (2002). Nunatak survival of the high alpine plant *Eritrichium nanum* (L.) Gaudin in the Central Alps during the ice ages. Molecular Ecology, 11, 2027–2036. 12296946 10.1046/j.1365-294x.2002.01595.x

[ece310227-bib-0096] Sturm, H. , & Machida, R. (2001). Archaeognatha. De Gruyter.

[ece310227-bib-0097] Tellier, A. (2019). Persistent seed banking as eco‐evolutionary determinant of plant nucleotide diversity: Novel population genetics insights. The New Phytologist, 221, 725–730. 30346030 10.1111/nph.15424

[ece310227-bib-0098] The UniProt Consortium . (2017). UniProt: The universal protein knowledgebase. Nucleic Acids Research, 45, D158–D169. 27899622 10.1093/nar/gkw1099 PMC5210571

[ece310227-bib-0099] Thorstensen, M. J. , Baerwald, M. R. , & Jeffries, K. M. (2021). RNA sequencing describes both population structure and plasticity‐selection dynamics in a non‐model fish. BMC Genomics, 22, 273. 33858341 10.1186/s12864-021-07592-4 PMC8048188

[ece310227-bib-0100] van Husen, D. (1997). LGM and late‐glacial fluctuations in the Eastern Alps. Quaternary International, 38–39, 109–118.

[ece310227-bib-0101] Wachter, G. A. , Arthofer, W. , Dejaco, T. , Rinnhofer, L. J. , Steiner, F. M. , & Schlick‐Steiner, B. C. (2012). Pleistocene survival on central alpine nunataks: Genetic evidence from the jumping bristletail *Machilis pallida* . Molecular Ecology, 21, 4983–4995. 22994297 10.1111/j.1365-294X.2012.05758.x

[ece310227-bib-0102] Westergaard, K. B. , Alsos, I. G. , Popp, M. , Engelskjøn, T. , Flatberg, K. I. , & Brochmann, C. (2011). Glacial survival may matter after all: Nunatak signatures in the rare European populations of two west‐arctic species. Molecular Ecology, 20, 376–393. 21156004 10.1111/j.1365-294X.2010.04928.x

[ece310227-bib-0103] White, M. J. D. (1973). Animal cytology and evolution. Cambridge University Press.

[ece310227-bib-0104] Ye, J. , Fang, L. , Zheng, H. , Zhang, Y. , Chen, J. , Zhang, Z. , Wang, J. , Li, S. , Li, R. , Bolund, L. , & Wang, J. (2006). WEGO: A web tool for plotting GO annotations. Nucleic Acids Research, 34, W293–W297. 16845012 10.1093/nar/gkl031 PMC1538768

