# Correction to “Evolutionary history of an Alpine Archaeognath (*Machilis pallida*): Insights from different variant”

**DOI:** 10.1002/ece3.10880

**Published:** 2024-01-25

Haider, M., Schilling, M. P., Moest, M. H., Steiner, F. M., Schlick‐Steiner, B. C., & Arthofer, W. (2023). Evolutionary history of an Alpine Archaeognath (*Machilis pallida*): Insights from different variant. *Ecology and Evolution*, **13**(7), e10227. https://doi.org/10.1002/ece3.10227

Within the title “Evolutionary history of an Alpine Archaeognath (*Machilis pallida*): Insights from different variant” there was an error. It should have read “Evolutionary history of an Alpine archaeognath (*Machilis pallida*): Insights from different variant types”.

We apologize for this error.

